# Associations of Meteorology with Adverse Pregnancy Outcomes: A Systematic Review of Preeclampsia, Preterm Birth and Birth Weight

**DOI:** 10.3390/ijerph110100091

**Published:** 2013-12-20

**Authors:** Alyssa J. Beltran, Jun Wu, Olivier Laurent

**Affiliations:** Program in Public Health, Anteater Instruction & Research Bldg (AIRB), 653 East Peltason Drive, University of California, Irvine, CA 92697, USA; E-Mails: ajbeltra@uci.edu (A.J.B.); olaurent@uci.edu (O.L.)

**Keywords:** preeclampsia, preterm birth, birth weight, meteorology, seasonality, climate, temperature, heat, cold, humidity

## Abstract

The relationships between meteorology and pregnancy outcomes are not well known. This article reviews available evidence on the relationships between seasonality or meteorology and three major pregnancy outcomes: the hypertensive disorders of pregnancy (including preeclampsia, eclampsia and gestational hypertension), gestational length and birth weight. In total 35, 28 and 27 studies were identified for each of these outcomes. The risks of preeclampsia appear higher for women with conception during the warmest months, and delivery in the coldest months of the year. Delivery in the coldest months is also associated with a higher eclampsia risk. Patterns of decreased gestational lengths have been observed for births in winter, as well as summer months. Most analytical studies also report decreases in gestational lengths associated with heat. Birth weights are lower for deliveries occurring in winter and in summer months. Only a limited number of studies have investigated the effects of barometric pressure on gestational length or the effects of temperature and sunshine exposure on birth weight, but these questions appear worth investigating further. Available results should encourage further etiological research aiming at enhancing our understanding of the relationships between meteorology and adverse pregnancy outcomes, ideally via harmonized multicentric studies.

## 1. Introduction

Adverse pregnancy outcomes are responsible for a considerable burden of morbidity and mortality worldwide, both in pregnant women and their offspring throughout their lifespan [1]. Among the most frequent and serious outcomes of pregnancy are hypertensive disorders, preterm birth and intrauterine growth retardation.

Hypertensive disorders during pregnancy occur in approximately 10% of pregnant women [2]. One of the most common is preeclampsia, a hypertensive syndrome specific to pregnancy, generally defined as new hypertension (blood pressure > 140/90 mm Hg) and substantial proteinuria (≥300 mg in 24 h) at or after 20 weeks’ gestation [3]. Preeclampsia may be associated with placental insufficiency and maternal organ dysfunction. It can also cause seizures, in the more severe form called eclampsia. Preeclampsia and eclampsia affect 2% to 8% of pregnancies worldwide and are major causes of maternal diseases, disability and death [2]. Preterm birth is defined as birth before 37 completed weeks of gestation. It is the major cause for infant death and may be responsible for infant and long-term cognitive function impairments, decreased motor functioning, increased behavioral disorders, impaired vision and hearing, respiratory complications, and substantial associated hospital cost and loss of school and work days [4]. More than 10% of pregnancies worldwide result in preterm births [4]. By hampering fetuses to complete their full intrauterine growth, preterm birth can result in infants born with a restricted weight. Yet another cause for restricted birth weight may be intrauterine growth retardation (IUGR), which is characterized by a small birth weight for gestational age. IUGR is associated with impaired child growth and increased risk of adult diseases in later life including type II diabetes, hypertension, and cardiovascular disease [5]. In 2010, approximately 11% of all infants were born with low birth weight (LBW, defined as below 2,500 g) worldwide [6].

There is a growing interest in the health effects of meteorology, especially since the frequency and magnitude of extreme meteorological events (e.g., heat waves, violent storms) are expected to increase in a context of climate change [7]. Meteorological conditions have been shown to influence several health outcomes, either communicable such as cholera, malaria and bacterial meningitis [8] or non communicable such as cardiovascular diseases [9]. The health effects of meteorological conditions might well extent to a broader set of outcomes, which would then be important to identify. Considering the frequency and impacts of adverse pregnancy outcomes, studying their relations with meteorological conditions appears of primary interest.

This systematic review aimed at synthesizing available evidence on the potential effects of meteorology on major pregnancy outcomes: the hypertensive disorders of pregnancy (including preeclampsia, eclampsia and gestational hypertension), gestational length (including preterm birth) and birth weight.

## 2. Material and Methods

### 2.1. Search Strategy

A comprehensive and systematic literature review was conducted of all original studies published in English that examined meteorological influences on the hypertensive disorders of pregnancy, preterm birth and birth weight. Human studies published between 1 January 1990 and 1 November 2013 were identified using the PubMed and Web of Science (ISI) databases to search for articles published in academic, peer reviewed journals. Standard Boolean logic was applied using the following format in PubMed: ((preeclampsia OR pre-eclampsia OR eclampsia OR pregnancy-induced hypertension OR gestational hypertension) AND (season* OR climate OR weather OR meteorology OR humidity OR precipitation OR rainfall OR barometric pressure OR atmospheric pressure OR sunlight OR temperature OR wind) AND (“1990/01/01”[PDat]: “2013/03/31”[PDat]) AND Humans [Mesh]). The same query was repeated for the other study outcomes: (preterm OR pre-term OR premature OR gestational length) and (birth weight OR low birth weight OR term birth weight OR small for gestational age). The same logic was applied for the search in Web of Science (ISI) except that no criterion was available to select only human studies.

### 2.2. Screening Process

Articles were retrieved individually for each outcome using a two step approach. First, titles and abstracts were screened for mentions of season, meteorological variables and pregnancy outcomes. Second, articles passing this first step were reviewed in depth to assess if they reported results for associations between at least one meteorological variable or seasonality and at least one of the pregnancy outcomes of interest. Reviews and duplicate publications were excluded since they did not report original findings. References of the retrieved papers were further examined to ensure that all relevant published papers were included.

The Pubmed and Web of Science database searches retrieved 173 and 169 articles, respectively, for the hypertensive disorders of pregnancy (35 of them meeting the above inclusion criteria), 1,774 and 3,085 for length of gestation including preterm birth (28 of them met the above inclusion criteria), and 1,080 and 2,246 articles for birth weight (27 of them met the above inclusion criteria).

### 2.3. Data Extraction

The following detailed information was obtained and tabulated according to outcome for each included study: summarized meteorological or seasonal variable; first author (year) and setting; climate classification; detailed definition of exposure metric of meteorological variable or seasonality; study design; inclusion criteria; statistical model; sample size; summarized results including effect sizes when available; and complementary information including adjustment for confounders. Base data were also extracted when they were suitable for calculating effect sizes for meta-analyses but that these effect sizes were not directly reported in publications.

### 2.4. Meta-Analyses

Whenever feasible, we computed quantitative summaries of available evidence using a meta-analysis approach. Meta-analysis was conducted whenever three or more studies met the following criteria and were pooled for a specific combination of outcome and exposure.

- They reported the same pregnancy outcome.
- They reported sample sizes.
- They examined the same type of exposure variable (e.g., two studies on temperature will be pooled but one study on temperature will not be pooled with another one on a heat-humidity index (composite variable based on temperature and humidity and calculated according to different formulae).
- They reported effect sizes estimates with consistent temporal resolutions, or at least provided base data of consistent temporal resolutions (e.g., by month or pregnancy trimester) allowing to compute effect size estimates.
- For month-to-month variations in pregnancy outcomes, pooling was conducted only for studies from locations showing comparable relative trends in month-to-month temperature changes (these temporal profiles were assessed from [10]). For studies conducted in the North hemisphere, this means December and January were the coldest months, and July and August were the warmest, with monotonic transitions in between. A 6-month lag was applied for studies conducted in the South hemisphere as compared to the North hemisphere. Meta-analyses were therefore conducted using a monthly indicator defined as follows: “January in North hemisphere OR July in South hemisphere”, “February in North hemisphere OR August in South hemisphere”, and so on for the other months.
- For season-to-season variations in pregnancy outcomes, we relied on the definitions of seasons provided by authors in their original publications. Some studies documented only month-to-month variations in pregnancy outcomes, and did not report effect size estimates by season. However, if these studies reported number of cases and total pregnancies by month, we aggregated monthly data to seasonal data and subsequently included them in the meta-analyses on season. The following were adopted for seasons in the North hemisphere: winter (December–February), spring (March–May), summer (June–August), and autumn (September–October). Again, a lag of 6 months was applied to define seasons in the Southern hemisphere.

Meta-effect sizes estimates and associated 95% credible intervals were calculated using random effects models allowing to account for heterogeneity in effect sizes estimates between different studies [11,12]. 

### 2.5. Presentation of Results

In the results section, we summarize the findings by pregnancy outcome. We first describe patterns reported by season of conception and season of birth, and then by specific meteorological variables such as temperature, humidity, precipitations sunshine or wind patterns. Meta-effect size estimates are presented whenever three or more studies of sufficiently homogeneous designs provided necessary data to conduct meta-analyses (as detailed in Section 2.4 above). Otherwise, results are presented using a narrative review approach.

In this summary, we separate studies conducted in tropical and non-tropical settings, defined by the Köppen-Geiger climate classification system [13], which divides climates into five main groups: tropical/megathermal, dry temperate, mild temperate, continental/microthermal, and polar. Articles were classified as either tropical, if they fell into any of the tropical subtypes (*i.e.*, tropical rainforest climate, tropical monsoon climate, or tropical wet and dry or savannah climate), or non-tropical if they did not. This distinction was made because many studies in tropical climates define season as rainy *vs*. dry, whereas seasons were generally defined as winter, spring, autumn and summer in the studies conducted in non-tropical settings.

## 3. Results

### 3.1. Findings from Hypertensive Disorders of Pregnancy

For hypertensive disorders of pregnancy, we retrieved 35 studies that examined three different pregnancy outcomes: preeclampsia (*n* = 24), eclampsia (*n* = 11) and gestational hypertension (*n* = 4).

#### 3.1.1. Preeclampsia

**Figure 1:**
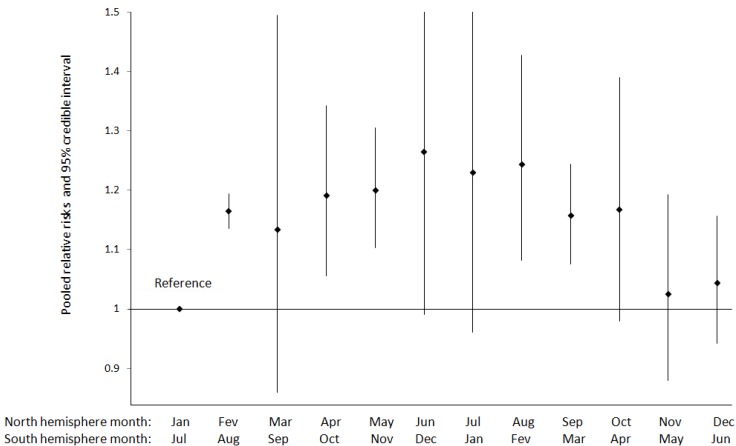
Pooled relative risks and 95% credible interval for the variation in preeclampsia incidence by month of conception (*N* = 530,160 births).

Six studies examining preeclampsia focused on seasonality of conception (Table A1). Five of them were conducted in non tropical settings and reported month-to-month variations that allowed meta-analysis [14,15,16,17,18]. The result of the meta-analysis including 530,160 births (Figure 1) shows an increase in risks of preeclampsia from the coldest to the warmest months of conception, followed by a decrease from the warmest to the coldest months of conception, although pooled relative risks were statistically significant only for certain months. One single study in Australia contributed to 80% of pregnancies included in the meta-analysis [16]. After excluding this study, we still observed a similar temporal pattern, although most relative risks are not significant anymore (Table A2). One study conducted in the tropical setting of Thailand could not be pooled with the other studies included in the meta-analysis, which were all conducted in non-tropical settings. This study reported a higher risk of preeclampsia for conception in dry than in wet season [19].

**Figure 2:**
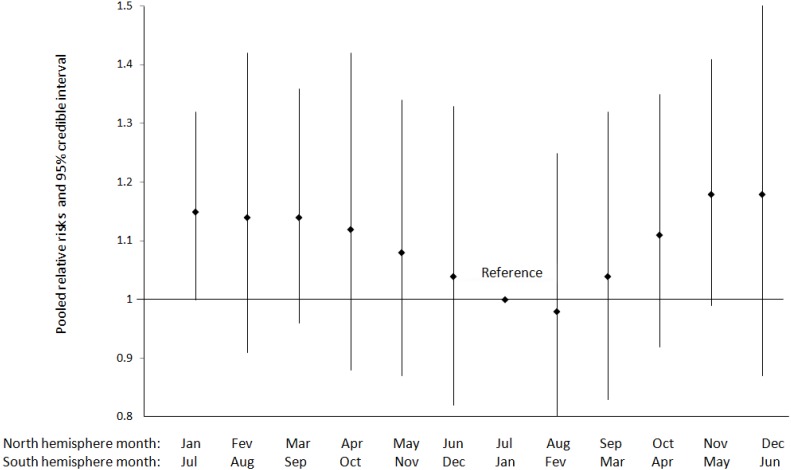
Pooled relative risks and 95% credible interval for the variation in preeclampsia incidence by month of birth (*N* = 2,552,887 births).

Among 19 studies focusing on seasonality of birth, 10 documented month-to-month variations (Table A3). Nine were conducted in non tropical settings and one in the tropical setting of Zimbabwe [20]. However, the monthly variations in temperature in Zimbabwe were judged sufficiently comparable with those of non-tropical settings to allow for a meta-analysis of 10 studies. The result of the meta-analysis including 2,552,887 births (Figure 2) shows a monotonic decrease in risks from the coolest to the warmest months of births, followed by an increase from the warmest to the coolest months of births, with significantly higher risk for the month of January/July (for the North/South hemisphere respectively) as compared to the month of July/January (for the North/South hemisphere respectively) but not for other months. This pattern is not affected by the exclusion of the sole tropical study [20] (data not shown). One study conducted in Norway accounted for 73% of all the pregnancies included in the meta-analysis [21]. The exclusion of this study led to less marked temporal pattern, and made the results insignificant (Table A4).

Additional meta-analyses on seasonality of birth were conducted by including studies documenting season-to-season variations (or month-to-month variations but with sufficient information to obtain seasonal aggregates). We pooled a different set of eight studies (three of which were also included in the above month-to-month analyses) with 386,839 births (Table A5). The highest pooled relative risks are observed for births in both winter and spring (summer being considered as a reference category, with the lowest risk), but results are not statistically significant (Table A6). A study conducted in Texas (USA) contributed to 80% of pregnancies [22] included in the meta-analysis. After the exclusion of this study, the highest rate ratio was observed in spring and was statistically significant, while summer still showed the lowest risk (Table A7).

Some studies on the seasonal variability of birth could not be pooled with others (Table A5): A study in Mississippi (USA) reported no significant difference of preeclampsia risk by season of birth based on only 3 seasons (spring, summer, autumn) [23]. Three studies conducted in tropical settings in India, Thailand and Zimbabwe reported no significant difference in preeclampsia risk between the monsoon and dry season [19,20,24]. However, not enough details were available at seasonal resolution [20] to allow for a meta-analysis. Last in Nigeria, the number of caesarians for preeclampsia was higher during the rainy than during the dry season; however this study did not consider any control population of non-preeclamptic women to allow for a comparison [25].

Ten studies focused on the association between preeclampsia and temperature or heat-humidity indices (Table A8). A study in Canada associated occupational exposures to extreme temperatures during the first 20 weeks of pregnancy with an increased preeclampsia risk. However, the definition of extreme temperature employed did not allow differentiation between cold and hot temperatures [26]. Another study in China found preeclampsia to be positively associated with higher heat index (defined as a function of temperature and humidity) at the time of conception with a lag of two months [18].

Three studies conducted in Israel, Kuwait, and South Africa found that the risk of preeclampsia was inversely associated with temperature during the month of birth [27,28,29]. From a slightly different set of three studies providing base data [28,29,30] we computed a pooled correlation coefficient between preeclampsia rates and mean temperature during the month of birth (an inverse but insignificant association was observed: R = −0.22, 95% credible interval: –0.71; 0.27). Two studies found no association between preeclampsia and mean seasonal temperature in Iran [31] and in the USA [23]. A study in Israel found that preeclampsia risk was associated with changes in daily overall differences of temperature exceeding 10 °C in any direction [30]. In tropical settings, only two studies in India [24] and Thailand [19] focused on the relation between preeclampsia and seasonal temperature and both found no significant difference in preeclampsia risk by. However, inter-seasonal contrasts in average temperature were low (<2 °C) in both settings.

Six studies examined humidity. Three studies conducted in non-tropical climates in Israel [27,30] and Kuwait [29] found that risk of preeclampsia was positively associated with high humidity during the month of delivery. However, these results could not be pooled since one study [27] only reports a contrast in preeclampsia rates above or below a 70% humidity threshold and similar indicator could not be reconstructed from the two other studies [29,30]. In the Mississippi (USA), no association between preeclampsia risk and mean seasonal humidity (calculated for three seasons) was observed [23].

In the tropical settings of India and Thailand, [19,24] no significant contrast in preeclampsia rates was observed between births in the rainy and the dry seasons, which saw 5%–10% contrasts in relative humidity and 200 mm [32] to 500 mm [24] contrasts in precipitations, respectively. However in Thailand, conception during the dry season was associated with an increased risk of preeclampsia [19]. Three more studies examined precipitations. A study in Zimbabwe observed increased preeclampsia incidence rates during months of delivery with high precipitations, but the results based on a dichotomous indicator for rainfall (15 mm threshold) was not significant [20]. In South Africa, no significant correlation was observed between monthly average rainfall and preeclampsia rates [28]. In Iran, precipitations averaged on each of four seasons were not associated with preeclampsia rates [31].

Results were scarcer for the association between preeclampsia and other meteorological parameters. One study in Australia found that increased sunlight exposure around conception was inversely associated with early onset, but not late-onset, preeclampsia [16]. One study in India found no association between barometric pressure during the season of delivery and risk of preeclampsia [24]. One study in Israel found that risk of preeclampsia was positively associated with number of days with strong winds (exceeding a speed of 5 m/s) [30]. 

#### 3.1.2. Eclampsia

**Figure 3:**
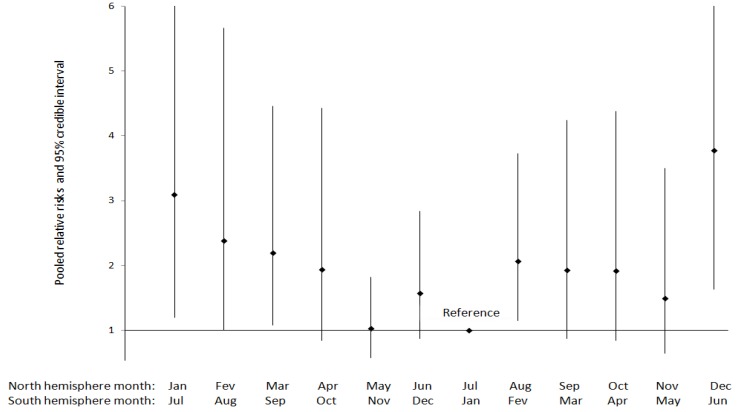
Pooled relative risks and 95% credible interval for the variation in eclampsia incidence by month of birth (*N* = 550,881 births).

No study examined eclampsia risk in relation to the time of conception. However, ten studies examining eclampsia focused on seasonality of birth (Table 1). Four studies were identified for the meta-analysis of month-to-month variations (Table A9), with three in non-tropical countries and one in a tropical setting in India [33]. However, the monthly variations in temperature in the tropical setting were judged sufficiently comparable with those of non-tropical settings. The result of the meta-analysis including 550,881 births (Figure 3) shows a decrease in risk from the coolest to the warmest months of births, followed by an increase in risk from the warmest to the coolest months of births, with significantly higher risks for the months of December/June to March/September as compared to the month of July/January (for the North/South hemisphere respectively). A significantly increased risk is also observed for the August/February month. If the largest study in Sweden (contributed 88% of pregnancies) [34] is excluded, these patterns remain although statistical significance is lost (Table A10).

Additional analyses on seasonality of birth were conducted by including studies documenting season-to-season variations (and studies with monthly data that can reliably be aggregated into seasonal variations) (Table A11). The meta-analysis (574,433 births) shows that the highest risk for eclampsia is observed for births in the winter, and it is significantly different from the risk in summer (Table A12). The pattern is similar, but statistical significance is lost, if the largest study from Sweden [34] (*N* = 482,759 births) is excluded (Table A13). Some studies could not be pooled with others: a study in India also reported increased risk of preeclampsia during the coldest months of the year, but did not mention sample size [35]. Preeclampsia cases were reported to be more common during the winter than during other seasons in Pakistan [36] and during the rainy than during the dry season in Nigeria [37], however these studies did not consider any control population of non-eclamptic women to allow for a comparison. Two studies conducted in the tropical settings of Ghana and India [24,38] found higher preeclampsia rates during the rainy than the dry season.

Six studies focused on the association between eclampsia and temperature but these could not be pooled because of missing sample size information [35], lack of control population [37] or heterogeneous temporal resolutions for temperature indicators. In a study in Pakistan, a significant positive association was observed between eclampsia and average temperature during the month of birth in one hospital, but not in three other hospitals [39]. Two studies in the tropical settings of India and Mozambique found an inverse association between risk of eclampsia and average monthly temperature [35,40]. Another study in India reported a significantly higher preeclampsia rate for birth during the rainy season characterized by slightly cooler temperatures [24]. One study in Iran reported no association between risk of eclampsia and average temperature during the season of delivery [31]. A study in Nigeria found a higher number of eclampsia cases in cooler than in warmer months [37], but no control population was considered for comparison.

Four studies focused on humidity [24,35,39,40] and three on rainfall [24,31,37]. No pooling was possible, for the same reasons as mentioned above for temperature. Two studies in Pakistan and Mozambique found no association between eclampsia and relative humidity during the month of birth [39,40], whereas a study in India reported a positive and significant association [35]. This last finding agrees with another Indian study that associated higher eclampsia risk with higher seasonal relative humidity and higher levels of rainfall [24]. However, a study in Iran identified no association between eclampsia and seasonal rainfall [31]. More eclampsia cases were observed during higher precipitation months in Nigeria [37], but no control population was considered for comparison.

Two studies examined sunlight in non-tropical climates, and findings were mixed. One study in Sweden observed inverse association between eclampsia rates and mean daily sunlight hours during the season of birth [34], whereas one study in Iran found no association [31]. Two studies in tropical settings (Mozambique, India) reported positive associations between eclampsia and low monthly or seasonal average barometric pressure [24,40].

#### 3.1.3. Gestational Hypertension

Only four studies, all conducted in non-tropical settings, examined gestational hypertension (GH) defined as new hypertension (blood pressure ≥ 140 mm Hg systolic and/or ≥90 mm Hg diastolic) arising after 20 weeks of gestation. One study in Australia reported that conception in spring was associated with an increased GH risk. GH was also positively correlated with solar radiation at one month after conception but inversely correlated with it at seven months after conception [16]. A study in Canada found no significant association between GH and occupational exposures to extreme temperatures at the onset of pregnancy [26]. Another study in Sweden found no significant differences in GH risk between seasons, although the highest rates were observed in winter [41]. Last, a study in Kuwait found higher GH risks for deliveries occurring in months of low humidity and high temperature [29]. Two studies documented the variation of blood pressure considered as a continuous variable in pregnant women. A study in the USA found that blood pressure declined steadily from January to August and rose August through December [42]. Consistently, one study in Japan found a 10 °C increase in daily minimum outdoor temperature reduced blood pressure by an average of 2.5 mmHg [43] (see Table 1).

**Table 1:** Associations between meteorology and the hypertensive disorders of pregnancy.

Reference	Seasonal or meteorological variable	Outcome	Setting (climate type ^a^), study period	Exposure metric	Study Design	Inclusion criteria	Statistical method	Population size	Summarized Main Results	Confounders adjusted for/ other comments
[27]	Seasonality of birth (month) Temperature Humidity	Preeclampsia	Tel Aviv, Israel (NT), 1984–1988	- Year divided into months: December–May (cooler), June–November (warmer) - Monthly average humidity and temperature	Hospital based cohort study	All women	unspecified	*N* = not stated but estimated to 18,500 using the average preeclampsia incidence. Preeclampsia cases: 276	- Statistically significant increase in incidence rates between January–June (1.5%) compared to July–December (1.15%) *p* < 0.035 - Statistically significant increase in incidence rates between December–May (1.6%) compared to June–November (1.1%), *p* < 0.001 -Incidence rates increased in months of low temperature and high humidity (>70%)	
[23]	Seasonality of birth (3 seasons) Temperature Humidity	Preeclampsia	Jackson, MS, USA (NT), January 1990–December 1992	- Year divided into seasons: spring (February–May), summer (June–September) and fall (October–January) - Daily maximum mean temperatures and relative humidity averaged by season	Hospital based cohort study	All women who delivered at a referral center	Chi square test	*N* = 11,958 cases: 995	- No association between preeclampsia and season of birth: Spring 7.8%; Summer 8.1%, and Fall 9.0% (*p* = 0.158). - No association of meteorological factors with mild preeclampsia *p* = 0.269), severe preeclampsia-eclampsia (*p* = 0.895), or superimposed preeclampsia (*p* = 0.193)	Maximum temperatures averaged by season were 73” F, 90.8” F and 65” F for spring, summer and fall respectively, whereas relative humidity was 85% , 89.7%. and 84%, respectively.
[29]	Seasonality of birth (month) Temperature Humidity	Preeclampsia Gestational hypertension	Safata, Kuwait (NT), 1992–1994	- Year divided monthly - Monthly average temperature and relative humidity	Hospital based cohort study	All women	Pearson correlation and linear regression	*N* = 28,262 Gestational hypertension cases: 1,457 Preeclampsia cases: 692	- No association between seasonal variation and preeclampsia or gestational hypertension - Preeclampsia cases peaked in November (temperature = 20.9 °C, humidity = 53.2) and were low in August (temperature = 37.7 °C, humidity = 19.5%) - Gestational hypertension rates were highest in June (temperature = 36.7 °C, humidity = 48.5%) and lowest in March (temperature = 18.6 °C, humidity = 48.5%).	
[44]	Seasonality of birth (month)	Preeclampsia	Tulsa, OK, USA (NT), January 2005–December 2007	- Year divided monthly	Hospital based cohort study	All women at least 18 years of age	ANOVA Contingency table analysis	*N* = 3,050 Preeclampsia cases: 176	Neither analysis of variance nor contingency table analysis revealed a significant seasonality of preeclamptic deliveries, (*p* = 0.94 and 0.95, respectively)	
[28]	Seasonality of birth (month) Temperature Rainfall	Preeclampsia	Cape Town, South Africa (NT), 2002–2003	- Year divided into seasons: summer (mid-December–March), autumn (mid-March–June), winter (mid-June–September) and spring (mid-September–December) - Daily minimum and maximum temperatures and daily rainfall averaged by month	Hospital based cohort study	All pregnancies	Logistic regression	*N* = 11,585 Preeclampsia cases: 1,329	Incidence highest in winter (13.6%) and lowest in summer (8.5%) (OR = 1.69, 95% CI: 1.07–1.53). Risk of developing pre-eclampsia in June higher than in February (OR = 2.81, 95% CI: 2.06–3.83). No significant correlation between rainfall and pre-eclampsia rates (r = 0.265, *p* = 0.405) Dose-response gradient according to minimum temperature: Tmin ≤ 8.5 °C: OR = 1.00 (ref) 8.5 < Tmin ≤ 10.5 °C, OR = 0.904, 95% CI: 0.764;1.068 10.5 < Tmin ≤ 12.5 °C, OR = 0.964 95% CI: 0.813; 1.144 12.5 < Tmin ≤ 14.5 °C, OR = 0.897 95% CI: 0.709; 1.119 14.5 < Tmin ≤ 16.5 °C , OR = 0.615 95% CI: 0.527; 0.717	
[20]	Seasonality of birth (month) Rainfall	Preeclampsia	Southern, Zimbabwe (T), January 1992–August 1995	-Year divided monthly -Mean monthly precipitation	Multi-hospital based cohort study including 3 hospitals: Mpilo Hospital Gwanda Hospital Beitbridge Hospital	All pregnancies	Kruskal-Wallis-Test	-Mpilo Hospital *N* = 40,456 Preeclampsia cases: 3538 -Gwanda Hospital *N* = 4,880 Preeclampsia cases: 50 -Beitbridge Hospital *N* = 5,870 Preeclampsia cases: 49	Preeclampsia rates increase at the end of the dry season and onset of rainy season with a statistically significant association for two out of three of the hospitals. Gwanda Hospital *p* = 0.077 Beitbridge Hospital *p* = 0.086 Mpilo Hospital *p* = 0.259 -Preeclampsia rates positively associated with mean monthly precipitation with incidence rates increasing during months of high precipitation	
[25]	Seasonality of birth (rainy *vs*. dry)	Caesarian for preeclampsia	Enugu, Southern Nigeria (T), 1996–2006	Rainy season (April–October) *vs*. Dry season (November–March)	Hospital based cohort study	All caesarean deliveries due to preeclampsia	N/A	1,579 caesarean deliveries	Among the eclamptics, 26 presented during the rainy season and four during the dry season (*p* < 0.05).	Rainy season runs from April to October (average rainfall 147–211 mm. The dry season extends from November to March (average rainfall 35–81 mm) No control population was considered for comparison
[45]	Seasonality of birth (4 seasons)	Preeclampsia	Zahedan, Iran, 2004–2007	Season (Spring, Summer, Autumn, Winter)	Case—control study	Age 15–45 years and gravid 1–3	Pearson’s correlation coefficient	2,488 cases *vs*. 2,488 controls	Pre eclampsia (%) Spring, 38.7 Summer, 41.9 Autumn, 58.6 Winter, 48.4	
[46]	Seasonality of birth (4 seasons)	Preeclampsia	Tehran, Iran, 2005–2006		Hospital based case-control study	Women over 35 or below 18 years of age, history diabetes, chronic hypertension and renal disease, any drug use, multi-fetal pregnancy, smoking, erythroblastosis fetalis, and non-Iranian were excluded	Logistic regression	318 pre-eclamptic *vs*. 318 control women	Odds ratio (95% confidence interval) Spring : reference Summer: 1.1 (0.7–1.6) Autumn: 0.8 (0.5–1.3) Winter: 2.1 (1.3–3.4)	
[26]	Extreme temperature	Preeclampsia Gestational hypertension	Quebec, Canada (NT), January 1997–March 1999	High temperature producing sweating in most people or low temperature obliging people to wear a coat, (never, rarely, often or always)	Case control	All women with singleton live births who had been employed since the first month of pregnancy, during at least 4 consecutive weeks and at least 20 h weekly, excluding those with several employments	Logistic regression	Preeclampsia cases = 102 Gestational hypertension cases = 92 Normotensive controls = 4,381	Increased risk of Preeclampsia when exposed to extreme temperature (OR = 1.6, 95% CI: 1.0–2.6) No significant increase in risk of Gestational hypertension when exposed to extreme temperature (OR = 1.3, 95% CI: 0.8–2.2)	Age, Parity, history of abortion, body mass index, smoking during the last 3 months of pregnancy, education, and leisure-time physical activity during the first trimester of pregnancy
[47]	Seasonality of birth (month)	Preeclampsia	Negev, Israel (NT), 1988–2007	-Year divided monthly	Hospital based cohort study	All singleton pregnancies	Time series Poisson regression	*N* = 203,462 Preeclampsia cases: 8,421	Incidence highest in the winter months with OR = 1.31 (1.18–1.46) in December, OR = 1.33 (1.19–1.48) in January. and OR = 1.38 (95% CI 1.24–1.54) in February *vs*. lowest in the summer months (with Aug. as the reference)	Ethnicity (*i.e.*, Jewish or Bedouin Arabs), maternal age, parity, gestational age at delivery, gender and birth weight
[30]	Seasonality of birth (month) Temperature Humidity Strong winds	Preeclampsia	Negev, Israel (NT), 1999	-Daily overall differences of temperature and humidity, duration of strong winds (speeds of ≥5 m per second)	Hospital based cohort study	All pregnancies	Time series Poisson regression	*N* = 11,979 Preeclampsia cases: 109	Preeclampsia rates increased with daily overall differences of temperature and humidity (*p* < 0.03), sharp variations in temperature with an average of 3-day lag (*p* < 0.003) and strong winds (*p* < 0.002)	
[22]	Seasonality of birth (4 seasons)	Preeclampsia	Texas, USA (NT), 2007	-Year divided into seasons: winter (December, January, Febember), spring (March, April, May), summer (June, July, August), and fall (September, October, November).	Hospital based cohort study	All initial cases	Logistic regression	*N* = 312,207 Preeclampsia cases: 12,418	-Odds ratios (95% CI) for delivery in the four season: Winter: reference Spring: 0.97 (0.92; 1.02) Summer: 0.96 (0.91; 1.01) Fall 0.94 (0.89; 0.99)	Maternal age, race, health insurance, co-morbidities
[21]	Seasonality of birth (month)	Preeclampsia (ICD-8 codes 637.4, 637.5, 637.6 , 637.7, 637.8 and 637.9)	Norway (entire country) (NT), 1967–1998	- Year divided monthly	Population based cohort study	All women	Logistic regression	*N* = 1,869,388 Preeclampsia cases: 51,801	Preeclampsia risk highest in winter months (October–January) with peak in December OR = 1.26, 95% CI 1.20–1.31) *vs*. August	Parity, maternal age, fetal gender, region, time period.
[41]	Seasonality of birth (4 seasons)	Preeclampsia (ICD-9 codes 642E and 642F) Gestational hypertension (ICD-9 codes 642D and 642X)	Uppsala, Sweden (NT), 1987–1993	- Year divided into seasons: winter (December–February), spring (March–May), summer (June-August), or fall (September–November)	Hospital based cohort study	All nulliparous women aged 34 years or less	Logistic regression	*N* = 10,659 Gestational hypertension cases: 10,666	Compared with winter, preeclampsia risk was significantly lower in summer (odds ratio = 0.68), *p* < 0.05. No significant association for gestational hypertension	Maternal smoking, age, height, education, place of birth, history of fertility, multiple pregnancy, type 1 and gestational diabetes, gender.
[48]	Seasonality of birth (month)	Preeclampsia (systolic blood pressure of 140–159 mm Hg and/or diastolic blood pressure of 90–109 mm Hg for the first time after 24 weeks of gestation and 2 random urine dipsticks of 1+ protein or 1 dipstick of 2+ protein)	USA (Boston, MA; Buffalo, NY; New Orleans, LA; New York–Columbia; Baltimore, MD; Richmond, VA; Minneapolis, MN; New York–Metropolitan; Portland, OR; Philadelphia, PA; Providence, RI; and Memphis, TN) (NT), 1958–1964	- Day 75 (16 March), day 150 (30 May), day 225 (13 August), and day 300 (27 October) chosen as representative days, with day 1 (1 January) as the referent.	Hospital-based cohort study from Collaborative Perinatal Project	All singleton pregnancies identified women as non-Hispanic white or non-Hispanic black, who gave birth from 20-45 weeks of gestation, without chronic hypertension or elevated blood pressure at 24 weeks of gestation	Logistic regression	*N* = 39,710 Preeclampsia cases: 1,350 (3.4%)	- In white women, incidence highest in winter months (November, December, and January), and lowest in summer months (July, August, September) with a trough in mid-Aug (*p* < 0.05). - In black women, no association between month of delivery and preeclampsia risk (*p* = 0.81).	Parity, race/ethnicity, smoking, maternal age, delivery year, marital status, study site, gestational age at delivery
[19]	Seasonality of birth and conception (rainy *vs*. dry) Temperature Humidity Rainfall	Preeclampsia	Bangkok, Thailand (T), 2008–2009	- Year divided into monsoon and dry seasons. - Daily mean maximum temperature, morning humidity and rainfall	Hospital based cohort study	Women without chronic hypertension, overt diabetes, renal or collagen vascular disease, or hyperthyroidism, history of irregular menstrual period within the past three months, or incomplete clinical data	Logistic regression	*N* = 7,013 Preeclampsia cases: 327	Women who conceived in the dry season at greater risk to develop preeclampsia than those who conceived in the monsoon season (5.3% *vs*. 3.7%, adjusted OR 1.51; 95% CI 1.18–1.93). Preeclampsia rates of women who delivered in both seasons were not significantly different: 5.0% in the dry season *vs*. 4.3% in the monsoon, *p* = 0.178	Age, parity, BMI, pre-pregnancy weight, pregnancy weight gain, gestational diabetes mellitus, smoking status Monsoon season significantly associated with humidity (77.0% *vs*. 68.7%,) and rainfall (196.5 mm *vs*. 37.0 mm), but not with mean maximum temperature (37.0 °C *vs*. 38.1 °C)
[14]	Seasonality of conception (month)	Preeclampsia (ICD-9 642.4, 642.5, 642.6 or birth record indication of preeclampsia and/or eclampsia)	WA (entire state), USA (NT), 1987–2001	Year divided monthly	Population based cohort study	Primiparous who gave birth to a singleton live infant; excluding those with pre-existing hypertension, renal disease, diabetes mellitus or missing date of conception	Multi-variable logistic regression	*N* = 79,298 Preeclampsia cases: 6,680	Lowest incidence among women who conceived during November (7.8%) and January (7.7%) and highest among those who conceived in April and July (both 8.9%). After adjustment, incidence rates significantly higher among women conceiving in February and April through August *vs*. January: 1.14–1.19).	Last Menstrual Period (LMP) year, maternal age, race/ethnicity, marital status, education, prenatal care timing, smoking, alcohol, weight at LMP
[15]	Seasonality of conception and birth (month)	Preeclampsia	Burlington, VT, USA (NT), 1 January 1995–1 July 2003	- Year divided monthly and seasonally in 3-month blocks based on conception and delivery	Hospital based Case control study	All women with singleton pregnancies	Logistic regression	*N* = 7,904 Preeclampsia cases: 142	No significant association of month (*p* = 0.2) of delivery with the risk of preeclampsia: winter 2.0%, spring 2.2%, summer 1.4%, fall 1.4% Significant association of month of conception (*p* = 0.003) with risk of preeclampsia with the highest risk in summer 2.3% (OR = 1.7; 95% CI 1.06, 2.75) compared with spring 1.4%, fall 1.7% and winter 1.6%.	Maternal age, race/ethnicity, fetal sex, diabetes mellitus, chronic hypertension, chronic renal disease, thrombophilia
[18]	Seasonality of conception (month) Heat Index (function of temperature and humidity)	Preeclampsia	Hong Kong, China (NT), 1995–2002	- Year divided into four seasons: spring (March–May), summer (June–August), autumn (September–November), winter (December–February) - Daily maximum temperature and relative humidity (Heat Index) averaged by month	Hospital based case control study	All singleton primiparous pregnancies excluding those with chronic hypertension, renal disease, pre-existing diabetes mellitus, or SARS	Logistic regression Cross correlation function	*N* = 15,402 Cases: 245	Conception during summer associated with a higher risk as compared with autumn (2.3 *vs*. 1.6%, OR 1.7, 95% CI 1.2–2.5) with highest rates in June (OR 2.8, 95% CI 1.5–5.2). - Monthly incidence of pre-eclampsia positively associated with heat index at the time of conception time lag by 2 months (r = 0.78, 95% CI 0.36–0.93).	Adjusted for maternal age and fetal gender
[16]	Seasonality of conception (month) Sunlight	Pregnancy induced hypertension (PIH) Early-onset preeclampsia (delivery by ≤34 weeks) Late-onset preeclampsia (delivery after 34 completed weeks)	New South Wales, Australia (NT), January 2000–December 2005	-Year divided into seasons: Winter (June–August), Spring (September–November), Summer (December–February), Autumn (March–May) -Monthly means of daily solar radiation	Hospital based cohort study	All singleton pregnancies but superimposed preeclampsia (on preexisting hypertension) excluded	Pearson correlation coefficients	*N* = 424,732 Pregnancy hypertension cases: 34,828 Preeclampsia cases: 11,902	- PIH rates lowest for autumn (7.3%) and highest for spring (8.9%) conceptions - Early-onset preeclampsia rates lowest for pregnancies conceived in November December (0.26%) and highest for pregnancies conceived in April (0.39%). - Late-onset preeclampsia rates lowest for conceptions in May/June (2.2%) and highest in October-February (2.6%). - PIH strongly and positively correlated (r = 0.67) with solar radiation at 1 month after conception. - Increased sunlight before delivery associated with decreased PIH (r = −0.67) - Sunlight around conception inversely associated with early onset preeclampsia (r = −0.51; *p* = 0.09). - No association between sunlight and late onset preeclampsia.	None
[24]	Seasonality of birth (rainy *vs*. dry) Temperature Humidity Rainfall Barometric Pressure	Preeclampsia Eclampsia	Mumbai, India (T), March 1993–February 1996	- Year divided into two seasons: Monsoon (June–August) Dry (September–May) - Daily temperature, humidity, rainfall and barometric pressure averaged by season	Hospital based cohort study	Only pre-registered patients, who have received antenatal care at hospital	Chi Square Mann-Whitney and Fisher’s Exact Test	*N* = 29,562 Preeclampsia cases: 1,238 Eclampsia cases: 34	- No association between meteorological and preeclampsia incidence and rates did not differ between the monsoon and the dry season (4.3% *vs*. 4.2%, *p* = 0.5, respectively) - Eclampsia incidence significantly higher in the monsoon (0.2% *vs*. 0.08%, *p* = 0.01).	Monsoon was cooler (median maximum temperature 30.7 °C *vs*. 32 °C, *p* = 0.01), more humid (median relative humidity 85% *vs*. 70%, *p* = 0.0008), and received higher rainfall (median 504.9 mm *vs*. 0.3 mm, *p* = 0.0002) than the rest of the year. Median barometric pressure during the monsoon (1,005 mb) significantly lower than the rest of the year (1,012 mb, *p* < 0.0001)
[31]	Seasonality of birth (4 seasons) Temperature Rainfall Sunlight	Preeclampsia Eclampsia	Rasht, Iran (NT), 1991–2001	- Year divided into seasons: (spring, summer, autumn and winter) - Mean seasonal temperature, rainfall, and hours of sunlight	Hospital based cohort study	All women referred to Gynecologic ward with a gestational age more than 20 weeks	Chi-square test	*N* = 12,142 Preeclampsia cases: 397 Eclampsia cases: 17	- No association between preeclampsia or eclampsia and season of birth although the highest rate of preeclampsia was in spring (3.6%), and the lowest rate was in summer (3%). - No association between meteorological variables and preeclampsia or eclampsia.	Parity, maternal age The mean temperatures of spring, summer, autumn and winter in these three years were 18.36, 25.56, 14.61 and 7.47 centigrade, respectively.
[37]	Seasonality of birth (rainy *vs*. dry) Temperature Rainfall	Eclampsia	Abuja, Nigeria (T), March 2000–March 2005	- Year divided into rainy season (late April–October) and dry season (November–early April) - Monthly average temperature & precipitation	Hospital based cohort study	All pregnancies	Monthly incidence comparison	*N* = 5,987 Eclampsia cases: 46	Thirty-one eclamptics (67.4%) admitted during the rainy season and fifteen (32.6%) during the dry season. Increased risk of eclampsia in the rainy season compared to the dry season. Greater risk of Eclampsia when temperature is low and rainfall is high	None No information is provided on the temporal distribution of control pregnancies
[40]	Seasonality of birth (month) Temperature Barometric pressure Humidity	Eclampsia	Maputo City, Mozambique (T), 1984	-Year divided monthly -Monthly averages of temperature, humidity and atmospheric pressure	Hospital based cohort study	All women	Linear regression	*N* = 37,469 Eclampsia cases: 70	Eclampsia incidence rates inversely associated with temperature (R = −0.78, *p* < 0.05) and atmospheric pressure (*p* < 0.001) Highest incidence for delivery in coldest months of June-August (0.31%); lowest in warmest months December-February (0.10%). No significant association with humidity	None
[35]	Temperature Humidity	Eclampsia	Hyderabad, India (T), September 1987–August 1988	Mean monthly temperature and relative humidity	Hospital based cohort study	N/A	Pearson’s correlation coefficient	N/A	Eclampsia incidence positively associated with humidity (R = 0.74, *p* < 0.01) and inversely associated with temperature (R = −0.77, *p* < 0.01)	
[49]	Seasonality of birth (4 seasons)	Eclampsia (“Severe preeclampsia”)	Thessaloniki, Greece (NT), 2008–2011	Season	Hospital based cohort study	Patients with mild preeclampsia or chronic hypertension were excluded	Unpaired *t*-test	*N* = 12,722 deliveries, including 94 “severe preeclampsia” cases	Higher incidence during the summer but not statistically significant (*p* = 0.12) Incidence: Summer 0.90% Autumn 0.67% Winter 0.61% Spring 0.76%	
[36]	Seasonality of birth (4 seasons)	Eclampsia	Peshawar, Pakistan (NT), 2007–2009	Season	Hospital based cohort study	Pregnant patients with other convulsive disorders and more than 7 days postpartum excluded	N/A	*N* = 23,000 including 108 eclampsia cases	Higher number of eclampsia cases in the winter: Autumn 17.59% Winter 34.25% Spring 26.85% Summer 21.29%	
[38]	Seasonality of birth (months) Humidity Rainfall Monthly variation	Eclampsia	Accra, Ghana (T), 1991	Amounts and number of days of monthly rainfall	Hospital based cohort study	All pregnancies managed at Korle Bu Teaching Hospital		*N* = 10,301, including 134 eclampsia cases	More cases of eclampsia in the months in which the rainfall was high and the relationship was more associated with the number of days of rainfall than the monthly amount of rainfall.	Patients had blood samples tested for malaria parasites and none was positive.
[17]	Seasonality of conception (4 seasons)	Preeclampsia	Urmia, Iran (NT), 2007–2008	Year divided into four seasons: spring, summer, autumn, winter	Hospital based cohort study	Single pregnancies, no history of hypertension, coagulative or renal disease or anti phospholipids syndrome	T-test, Fisher test, Chi Square test	*N* = 2,824 *n* = 166 preeclampsia cases	- Preeclampsia incidence was higher following conception during warm seasons (spring and summer) *p* = 0.038 - temperature at conception non significantly higher in preeclampic women	
[39]	Seasonality of birth (month) Temperature Humidity	Eclampsia	Karachi, Rawalpindi, Peshawar, and Quetta, Pakistan (NT, T), 1996	- Year divided monthly by region -Mean monthly temperature and humidity	Multi-hospital based cohort study of 4 hospitals: Jinnah Post Graduate Medical Centre, Sandeman Hospital Quetta, Holy Family Hospital, Lady Reading Hospital Peshawar	All pregnancies	Pearson’s correlation coefficients	*N* = 18,483 Eclampsia cases: 395	- Jinnah Post Graduate Medical Centre: Eclampsia rates highest in summer months April–September. Eclampsia rates not significantly correlated with temperature (r = 0.21). - Sandeman Hospital Quetta: Eclampsia rates highest in winter months with two peaks in May and August. Eclampsia rates not significantly correlated with temperature (r = 0.03). - Holy Family Hospital: Eclampsia rates highest in summer (May–September) Eclampsia rates positively correlated with temperature (r = 0.74, *p* < 0.01). - Lady Reading Hospital Peshawar: Eclampsia rates highest in winter months as well as summer with a peak in May–July. Eclampsia rates not significantly correlated with temperature (r = 0.42) - No association with humidity	None
[34]	Seasonality of birth (4 seasons) Sunlight	Eclampsia	Sweden (NT), 1990–1994	- Year divided into winter (December–February), spring (March–May), summer (June–August) and autumn (September–November) - Mean daily hours of sunlight	Population based cohort study	All singleton pregnancies	Logistic regression	*N* = 482,759 cases: 182	Incidence of eclampsia nearly doubled during the winter season as compared to other seasons (reference). Summer OR 1.1; 95% CI (0.7–1.7) *vs*. winter OR 1.9 95% CI (1.3–3.0). - Eclampsia rates inversely associated with sunlight hours	Smoking, maternal age, parity, region and fetal gender.
[33]	Seasonality of birth (month and season)	Eclampsia	Jodhpur, India (T), January−December 2001	Month Season (winter from December to February, Dry summer from March to May, Monsoon from June to September and Post monsoon from October to November)	Hospital based cohort study	All women delivering at hospital during study period	Incidence rate and incidence ratio	*N* = 12,170 *n* = 197 eclampsia cases	- Incidence of eclampsia was highest in monsoon season (2.05%) and winter season (1.70%) (*p* = 0.048) and a minimum in summer (1.22%)	None
[43]	Temperature, Atmospheric pressure Humidity	Blood pressure	Miyagi Prefecture, Japan 2006–2007	Daily minimum, maximum and mean outside temperatures, daily mean atmospheric pressure, relative humidity and duration of sunshine	Panel study	Healthy pregnant women	Linear mixed model with individuals as a random effect.	101	A 10 °C increase in daily minimum outdoor temperature reduced blood pressure by an average of 2.5 mmHg Atmospheric pressure positively and humidity inversely associated with blood pressure. No association with sunshine	Gestational age When two meteorological parameters were entered into the same model, only the effect of daily minimum outside temperature remained significant
[42]	Seasonal variation	Blood pressure	Pittsburgh, PA (USA) 1997–2001	Month of measurement	Hospital base panel study	Women without spontaneous abortion, ectopic pregnancy or other adverse event, chronic hypertension , another pre-existing medical complication (*n* = 17), unknown pregnancy outcome, multiple gestation	Generalized estimating equations	1,919	Blood pressure declined steadily from January to August and rose August through December. After adjusting for gestational age, year, prepregnancy BMI, race, and multiparity, systolic blood pressure was 1.0 to 1.7 mm Hg higher from January to May, 0.6 mm Hg higher in September and October, and 0.8 mm Hg higher in November and December compared with August. Similar but not statistically significant seasonal trends were seen in diastolic blood pressure	Gestational age, year, prepregnancy BMI, race, and multiparity,

^a^ NT: Non-tropical climate; T: Tropical climate.

### 3.2. Findings from Length of Gestation Including Preterm Birth

We identified 28 studies that examined different pregnancy outcomes related to length of gestation (Table 2): mean gestational length (*n* = 7), preterm birth (*n* = 19), the onset of labor (*n* = 5) and premature rupture of membranes (*n* = 3). A large study conducted in the USA (*N* = 1,435,213 births) found lower mean gestational length for conceptions during the first months of the year, with a sharp minimum for May conceptions [50]. Although the outcomes differ, this is compatible with another USA study showing the highest preterm birth rates for infants conceived in March and May [51]. Besides, when the three available studies focusing on conception season and preterm birth are pooled (562,852 births) (Table A14), the highest pooled relative risk, although not statistically significant, is observed for conception in spring (Table A15). 

Four studies examined mean gestational length in relation with the time of birth. No pooling was feasible between these four studies, because of the important differences in the definition of seasons between one study [52] and the others [53,54,55] and the lack of sample size information in another study [55]. One study in Japan reported that infants born during the winter and summer seasons had shorter gestational lengths [53] than those born in spring or autumn. A Danish study reported gestational ages of winter-born infants were on average one day shorter than that of infants born in other months [55]. A Greece study reported that births in spring or summer had gestational ages about 4 days shorter than those in autumn or winter [54]. Lastly, in Zimbabwe infants born in the early dry season had gestational ages two to three weeks shorter than those born in the late rainy season [52].

**Figure 4:**
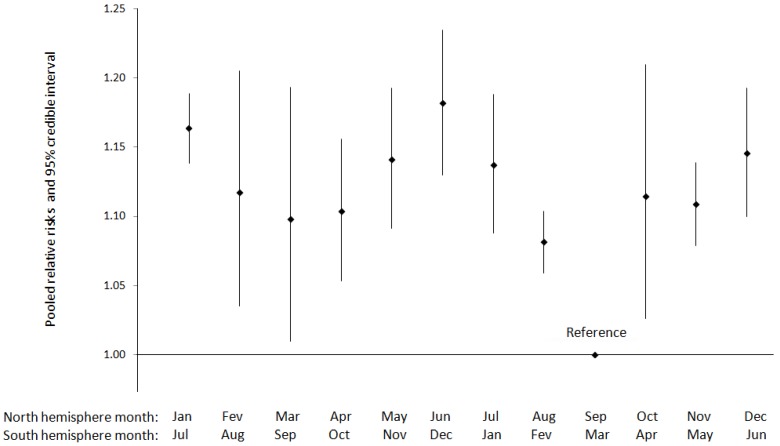
Pooled relative risks and 95% credible interval for the variation in preterm birth incidence by month of birth (*N* = 63,227,292 births).

Six studies focused on the variations of preterm birth risk by month of birth. Five of them, all conducted in the North hemisphere, met the criteria for a meta-analysis and contributed 63,227,292 births (Table A16). The pooled relative risks show two peaks of preterm births during the winter months (maximum in January) and the beginning of summer (maximum in June) (Figure 4). Even if the largest study conducted in the USA (82% of births in this meta-analysis) [56] is removed these two peaks are still observed (Table A17).

For meta-analysis by four seasons of birth with a total of 11,703,114 births (Table A18), no significant difference is observed between seasons, although the lowest relative risks are observed for births in spring and autumn (Table A19).

Some studies were not eligible in the above meta-analyses. In Canada, an increased risk of preterm birth was observed during an ice storm season [57]. One study in the Gambia identified two peaks of preterm birth incidence in July and October, and an increased risk of preterm birth in the rainy season as compared to the dry season [58]. In Zimbabwe, infants born in the early dry season were significantly more likely to be preterm than those born in the late rainy season [52], whereas in Indonesia no difference in preterm birth risk was found between the dry and the rainy season [32].

Three studies focused on the associations between temperature and mean gestational length. Pooling them was not possible because of differences in the definitions and temporal resolution of temperature indicators. One large study in Greece (516,874 births) found that average temperature during the month of birth was inversely associated with mean gestational age [54]. A recent study of 7,585 births in Spain reported an inverse association between daily heat-humidity index and mean gestational age, with effects lagged by up to five days [59]. However a study of 11,972 births in the USA during a period of heat wave (June–August 1995) detected no association between daily temperature and mean gestational length [60], after examining effects lagged by up to three days.

Nine studies focused on the associations between temperature and preterm births [61,62,63,64,65,66,67,68,69]. No meta-analysis was feasible because of differences in the definitions and temporal resolution of temperature indicators. Six of them reported positive associations between increases in temperature and the risk of preterm birth. A large study in Japan (7,675,006 births) reported that rates of preterm births and monthly average temperature were inversely correlated (R = −0.424, *p* = 0.003) in the winter, but positively correlated in the summer (R = 0.549, *p* < 0.001) [61]. A study of 11,979 births in Israel found that preterm birth rates increased as monthly average maximum temperature increased [64]. In these two studies, temperatures were averaged based on all the days of the month of birth whether these days preceded or were subsequent to births. The influence of temperature during the exact four weeks preceding birth was examined in an Australian study of 101,870 births, which reported a positive association with preterm birth between 28 and 36 gestational weeks. A small USA study (3,972 births) reported no significant association between a heat-humidity index averaged on the week of birth and preterm birth, but a positive association between the heat-humidity index and preterm labor [67].

Other studies focused more precisely on the potential influence of temperature in the week and the few days preceding birth. One study of 291,517 births in Germany reported no association between preterm birth and temperature in the last week preceding delivery [66] or during the first month or trimester of pregnancy. An even larger study of 482,765 births in England reported no association between the risk of preterm birth and temperature in the day of, or 6 days preceding birth [65]. However, temperatures were mild in these two settings [66,70] which did not allow exploring the effects of extreme temperatures. In California (USA) that experiences hotter temperatures, a study found a positive association between apparent temperature and preterm births, with effects lagged by up to six days [63]. A study of 132,691 births in Italy reported a positive association between apparent temperature during the warm season and increased risk of preterm birth [69]. One study of 154,785 births in Australia examined the association between preterm birth and heat waves [68]. Risks for of preterm births increased by 13% to 100% depending on the heat wave definition (see Table 2). Last, a small study of 1,088 births in the USA failed to identify temperature as a significant predictor of onset of labor in term or preterm infants [71]. In summary, eight out of twelve studies reported a positive association between temperature and preterm birth or mean gestational length.

Five studies examined barometric pressure [65,71,72,73,74]. One large study conducted in England (482,765 births) [65] reported no association of preterm birth with daily mean barometric pressure, or with the largest daily drop in barometric pressure.

Three studies focused on labor onset [72,73,74], but could not be pooled because of the heterogeneity in the definition of categorical variables for barometric pressure. A small USA study (162 births) reported a significantly higher occurrence of labor onset in the day following, than preceding a drop in barometric pressure (defined as ≥0.06 inches of mercury in 24 h) [74]. Another USA study of 2,435 births reported no significant difference in the frequency of labor onset between days in the lower tertile of daily mean barometric pressure versus the two other (higher) tertiles, although a significant decrease in the frequency of labor onset was observed after 3 consecutive hours of falling barometric pressure [73]. A study of 2,278 births in Japan reported no differences in the frequency of labor onset, whether barometric pressure was above or below a threshold of 1,011 hPa, however an increase in the frequency of rupture of the membranes was associated with barometric pressure below that threshold [72]. A small USA study (1,088 births) jointly studied labor onset and rupture of membranes as a single outcome and reported no significant association with hourly barometric pressure [71].

Fewer results were available for other meteorological parameters. Studies conducted in England [65] and the USA [71] reported no association between preterm birth and humidity. However a study in Israel found preterm birth risk to be positively associated with sharp increases in relative humidity and with strong winds (wind speed > 6 m/s) [64] (see Table 2).

**Table 2:** Associations between meteorology and the length of gestation, including preterm birth.

Reference	Seasonal or Meteorological Variable	Outcome	Setting (climate type ^a^), study period	Exposure metric	Study Design	Inclusion criteria	Statistical method	Population size	Summarized Main Results	Confounders adjusted for/other comments
[64]	Seasonality of birth (month) Temperature Humidity Wind	Preterm birth (<37 gestational weeks) Preterm premature rupture of membranes	Negev, Israel (NT), 1999	- Monthly mean relative minimum humidity and mean daily overall differences of relative humidity - Monthly mean temperature and daily overall differences of temperature - Number of days with strong winds	Retrospective cohort study	All deliveries before 37 completed weeks of gestation	Time series Poisson regression	*N* = 11,979 992 (8.3%) preterm 862 (7.2%) were complicated with PPROM	Preterm birth incidence: highest in June and December and positively associated with monthly mean relative humidity and maximum temperature (*p* < 0.01). - Positively associated with semiannual (*p* < 0.02) and seasonal (*p* < 0.05) variations of windIncreased rates of preterm delivery preceded sharp variations of relative humidity and maximum temperature by 3 days (*p* < 0.01).	
[57]	Seasonality of birth (according to period of ice storm)	Preterm birth (<37 gestational weeks)	Québec, Canada (NT), 1993–2003	Three periods (1993–1997, 1998 and 1999–2003, the referent period) corresponding to intervals prior to the storm, the year of the storm, and well after the storm	Population based study	Singleton live born infants from the Québec birth file; gestational age is confirmed with ultrasound examinations	Logistic regression	*N* = 855,320	- 28% higher odds of preterm birth for 1998 relative to 1999–2003 in areas affected by an ice storm	Region, time period, education level, maternal age, marital status, parity, maternal birth place
[69]	Temperature Heat waves	Preterm birth (early preterm (22–32 weeks) and late preterm (33–36 weeks))	Rome, Italy (NT), 2001–2010	- Daily maximum apparent temperature (MAT, index including both air and dew-point temperatures) during the warm season (April–October) - Daily minimum temperature (TMIN) in the cold season (November–March) lag 0–2 days selected for final analysis (from up to 30 days) - Heat waves (at least two consecutive days with MAT above the monthly 90th percentile or TMIN above the monthly 90th percentile and MAT above the median monthly value)	Hospital based cohort study	Exclusion of multiple births, all cesarean sections where spontaneous onset of labor was not reported, labor inductions, births referred with congenital malformations, and stillbirths, mothers younger than 11 years or older than 55 years	Times series analysis (Poisson generalized additive model conducted separately for cold and warm Seasons)	132,691 births, 7,259 (5.5%) of which were preterm	For the warm season: - increase of 1.9% (95% confidence interval 0.86–2.87) in preterm births per 1 °C increase in maximum apparent temperature in the 2 days preceding delivery - increase of 19% (95% CI 7.91–31.69) in preterm births during heat waves During the cold season, temperature had no significant effect When stratifying the analysis by gestational-week categories, the effect of temperature was only significant for late preterm births (late preterm: 1.93% change, 95% CI 0.88; 2.98; early preterm: −1.02% change, 95% CI −2.46; 45)	Long term trend, seasonality holiday, influenza in winter, particles with aerodynamic diameter of 10 µm or less, ozone, and nitrogen dioxide in the month preceding delivery No modification of the temperature effect by ozone was observed
[68]	Heat wave	Preterm birth (<37 weeks of gestation)	Brisbane, Australia (NT), 2000–2010	- 9 definitions of heat waves according to combinations of daily maximum temperature exceeding the 90th, 95th, and 98th percentiles of daily maximum temperature distribution of the study period for at least 2, 3, or 4 consecutive days during the last gestational weeks before delivery.	Population based study using birth certificates	Spontaneous singleton live births Warm season (between November and March) and cold season (November–March)	Cox-proportional hazards model	*N* = 154,785 including 50,848 preterm	- Hazard ratios of preterm birth ranged from 1.13 (95% CI: 1.03–1.24) to 2 (95% CI: 1.37–2.91) compared to women unexposed to at least one heat wave in warm season Results changed to some extent when different air pollutants were added into the model separately	Particulate Matter < 10 µm in diameter, nitrogen dioxide, ozone, carbon monoxide Sex and weight of baby, onset of labor (spontaneous, induced, and caesarean), mother’s residential area (postcode), maternal age, marital status, indigenous status, parity, year, month neighborhood socioeconomic level
[63]	Temperature	Preterm birth (<37 gestational weeks)	CA (16 counties), USA (NT), May–September 1999–2006	- Daily mean, maximum and minimum apparent temperature during the warm season (1 May to 30 September)	Population-based cohort of 16 counties	All cases of preterm birth from a state registry of births	Case-crossover (logistic regression)	*N* = 58,681	- Significant positive association between apparent temperature and preterm birth with effect estimates significantly elevated for up to 6 days lags, a weekly average of apparent temperature being the best predictor. - No association for full-term births (37–44 weeks)	Air pollutants: Particulate matter with aerodynamic diameter <2.5 mm , ozone, nitrogen dioxide, carbon monoxide, and sulfur dioxide
[66]	Seasonality of conception and birth (4 seasons) Temperature	Preterm birth (<37 gestational weeks)	Brandenburg, Germany (NT), 2002–2010 Saxony, Germany (NT), 2005–2009	- Year divided into seasons: winter (December–February), spring (March–May), summer (June–August), autumn (September–November) - Daily mean temperature averaged on the first month, first trimester and last week of pregnancy	Time series analysis	All singleton births ≥ 20 weeks and ≤ 37 weeks of gestation with birth weight greater than 200 g	Time series logistic regression Fourier series	Brandenburg *N* = 128,604, including 8,717 preterm Saxony *N* = 162,913, including 10,277 preterm	- Weak association between preterm birth and conception in spring in Brandenburg (OR = 1.08, 95% CI 1.01–1.15) - Weak association between preterm birth and season of birth in winter in Saxony (OR = 1.07, 95% CI 1.01–1.13) - No association between preterm birth and temperature in Brandenburg in first trimester (OR = 0.94, 95% CI 0.85–1.04), second trimester (OR = 0.97, 95% CI 0.84–1.12), or third trimester (OR = 1.00, 95% CI 0.93–1.08) - No association between preterm birth and temperature in Saxony in first trimester (OR = 1.03, 95% CI 0.94–1.14), second trimester (OR = 1.06, 95% CI 0.94–1.21), or third trimester (OR = 1.00, 95% CI 0.94–1.07)	Maternal age available for Saxony only No adjustment was made for air pollution, hypertensive disorders of pregnancy or infections.
[75]	Seasonality of conception (4 seasons)	Preterm birth (<37 gestational weeks)	NC (entire state), USA (NT), 2001–2005	Year divided into seasons: winter (December–February), spring (March–May), summer (June–August), and fall (September–November)	Retrospective cohort study	Singleton first births to non-Hispanic white and black women, excluding births with missing covariate data, congenital anomalies, birth weight < 400 g, extreme gestational age, and maternal age > 44 years	Logistic regression	*N* = 188,276	Spring conceptions had the highest rates of preterm birth among non-Hispanic white births (*p* < 0.05). Among non-Hispanic black summer conceptions had the highest rate of preterm birth (*p* < 0.05).	Maternal age, education level, marital status, smoking status, region of North Carolina, county urbanization
[76]	Seasonality of birth (month)	Preterm birth (<37 gestational weeks)	Japan (entire country) (NT) January 1979–1983	Seasons: spring (March–May), summer (June–August), autumn (September–November), and winter (December–February). -rainy season (June–July) and typhoon season (August–October)	Retrospective cohort study	N/A	Time series regression	*N* = 7,665,006	Preterm, term, and post term all have a similar appearance with two peaks in winter and summer (or rainy season), and with two troughs in spring and autumn.	
[51]	Seasonality of conception (month)	Preterm birth (<37 gestational weeks) Very preterm birth (<32 gestational weeks)	Pittsburgh, PA, USA (NT), 1995–2005	Conception date was grouped by month of the year or by season: winter (December, January, February), spring (March, April, May), summer (June, July, August) and autumn September, October, November)	Retrospective cohort hospital based study	All births with available length of gestation information were included	Fourier series	*N* = 83,059	-Preterm birth associated with conception season (*p* < 0.05). Peak incidence occurred in winter and spring an average trough among late summer/early autumn conceptions -Similar pattern for very preterm birth (*p* < 0.05)	Parity, race/ethnicity, smoking, maternal age, delivery year, marital status, study site,
[61]	Seasonality of birth (month) temperature	Preterm birth (<37 gestational weeks)	Japan (entire country) (NT), 1979–1983	Month of birth Monthly mean temperature	Retrospective cohort study	N/A	Time series, Box-Jenkins autoregressive integrated moving average model	*N* = 7,675,006	For Japan the seasonality shows two peaks in the summer and the winter. The winter peak is most prominent in the North, the winter peak most prominent in the South The average risk of preterm births in winter is inversely correlated with mean temperature (r = −0.424, *p* < 0.01) The average risk of preterm births in summer is positively correlated with mean temperature (r = 0.549, *p* < 0.01)	Average maternal age, subsequent/first birth ratio, infant mortality rate, total fertility rate, population density, prefectural per capita income, and number of hospital beds and doctors per 100,000 inhabitant
[32]	Seasonality of birth (rainy/dry)	Preterm birth (<37 gestational weeks)	Lombok, Indonesia (T), 2001–2004	Rainy season (November–March)	Double blind cluster randomized controlled trial	All singleton live births with birth weight measured within 72 h of birth	Hierarchical logistic regression	*N* = 14,040	No significant association between preterm birth and the rainy season, *p*-value = 0.14, OR = 0.94, (0.87–1.01)	Infant’s sex, mothers’ residence, nutritional status, education, household wealth, mid-upper arm circumference, height, birth order and pregnancy interval
[58]	Seasonality of birth (month)	Preterm birth (<37 gestational weeks)	Keneba, Manduar, and Kantong Kunda, The Gambia (T), 1976–2003	-Year divided monthly	Retrospective cohort study	All live births in 3 subsistence-farming villages of the West Kiang District	Fourier series	*N* = 1,916	Preterm birth showed 2 peaks—in July (17.2%) and October (13.9%)	Malarial infection, maternal workload
[54]	Seasonality of birth (4 seasons) Temperature	Preterm birth (<37 gestational weeks) Gestational Length (continuous variable)	Greece (entire country) (NT), 1999–2003	-year divided into four seasons: winter (December–February), spring (March–May), summer (June–August), and autumn (September–November) -mean air temperature during birth month	Retrospective cohort study using birth registries	All Greek citizens born between the years 1999-2003 and all Greek citizens who died between the period.	General log-linear regression	*N* = 516,874	Incidence rates of fetal growth restriction and premature birth statistically lower (*p* < 0.05) for infants born during the autumn and winter than other seasons. -Mean ambient temperature during the month of birth in the infant database inversely correlated with gestational age (r = −0.22, p < 0.001)	Bonferroni correction
[56]	Seasonality of birth (month)	Preterm birth (<37 gestational weeks)	USA (entire country) (NT, 1989– 2001)	Year divided into months:	Population-based cohort study	All birth certificates included	Linear regression	52,041,052	Early spring and late summer births are less likely to be premature	
[52]	Seasonality of birth (rainy/dry)	Preterm birth (<37 gestational weeks) Gestational length (continuous variable)	Harare, Zimbabwe (NT), 1996–1997	Year divided into seasons: early (June–August) and late (September–November) dry, and early (December–February) and late (March–May) rainy	Randomized, controlled multi-micronutrient trial	All women between 22 and 36 weeks gestation	Linear regression	*N* = 1,669	Those born in the early dry season had a 2·3 (95% CI: 1·7; 2·8) weeks shorter gestation than those born in the late rainy season. Those born in the early rain season had a −0.8 (95% CI: −1.3; −0.3) weeks shorter gestation	HIV infection, malaria parasitaemia
[59]	Heat Index (function of temperature and humidity)	Gestational length (continuous variable)	Barcelona, Spain (NT), 2001–2005	Three daily indicators of extreme values of heat index (HI) percentile 90, 95 and 99)	Retrospective hospital cohort study	All deliveries excluding multiple births (*n* = 150), elective (*n* = 553) and emergency (*n* = 282) cesarean sections, labor inductions (462), and mothers referred for obstetrical pathology (*n* = 159)	First stage: a dynamic model was fitted to predict log-transformed region wide monthly average of gestational age Second stage: linear regression	*N* = 7,585	- non significant reduction (0.2 day) in average gestational age associated with an HI95 episode on the day of delivery –HI90 episode on the day before delivery associated with a 1-day reduction in average gestational age -more extreme HI95 episode on the day before delivery associated with a 2-day average gestational age reduction, - most extreme condition (HI99) associated with a 5-day average gestational age reduction	Ethnicity, maternal education level, parity, maternal history of preterm birth, use of assisted reproductive technique, maternal infection, maternal age, smoking status, occupational status, maternal diabetes, maternal obstetrical-gynecological pathology, infant sex
[50]	Season of conception (month)	Gestational length (continuous variable)	NJ (entire state), USA (NT), 1997–2006, New York, NY, USA (NT), 1994–2004 PA (entire state), USA (NT), 2004–2010	Month of birth	Population based cohort	Single births with nonmissing information on gestation length	Cohort study based on comparison between siblings	*N* = 1,435,213	The gestation length decreases from conception in January to May and jumps back to the January level for conception in June. A May decrease in gestation length by 0.8 wk leads to a 13% increase in premature births	Stable maternal characteristics (by design) Influenza Strong correlation of gestation length and the prevalence of influenza (the correlation coefficient is −0.71)
[71]	Temperature, Humidity Barometric pressure	Labor onset or premature rupture of membranes (time of parturition)	Evanston, IL, USA (NT), summer, fall, and winter of 2001	- hourly barometric pressure, temperature, and humidity (with lags of 0, 1 or 2 days)	Retrospective hospital cohort study	All patients delivering at hospital after spontaneous labor or rupture of membranes at ≥20 weeks of gestation	Logistic regression. E	*N* = 1,088	None of the individual weather variables identified as a statistically significant predictor of labor onset or premature rupture of membranes	Maternal age, gestational age, parity, multiple gestation and intrauterine infection
[60]	Temperature	Gestational length (continuous variable)	Illinois, USA (NT), June–August 1995	- daily maximum apparent temperature during the warm season (June–August) 0-, 1- and 2-day lag explored	Population-based cohort study	All singleton vaginal births	t tests for difference in means between categories of maximum apparent temperature: <90° F, 90 to 99° F, 100 to 109° F ≥ 110° F	*N* = 11,972	No evidence that increasing maximum apparent temperature was associated with shortened gestation length.	Maternal race/ethnicity, educational status and community area median household income
[53]	Seasonality of birth (month)	Gestational length (continuous variable)	Japan (entire country) (NT), January 1974–December 1983	- Year divided into seasons: spring (March–May), summer (June–August), autumn (September–November), and winter (December–February).	Time series analysis	All live singletons	Time series, analysis of variance	*N* = 16,796,415	Seasonal heterogeneity of mean gestational period (*p* < 0.001): two peaks for infants born in October and February–March, a deep trough in winter and a smaller one in June–September	
[55]	Seasonality of birth (one statement on winter month VS rest of the year)	Gestational length (continuous variable)	Denmark (NT), 1973–1994	- Year divided monthly with two adjoining 10-year secular trends as independent variables.	Population based cohort study	All children born within study period	Linear regression analysis	*N* = 1,166,206	Gestational ages of children born in December, January, and February were on average 1 day shorter than for children born in other months	
[65]	Temperature Humidity Precipitation Sunlight Barometric pressure	Preterm birth (<37 gestational weeks)	London, UK (NT), 1988–2000	-Year divided monthly -Daily temperature, rainfall, sunshine, relative humidity, barometric pressure, and largest drop in barometric pressure Cumulative exposure from 0 to 6 days before births explored	Time series analysis	All infants excluding those born before 24 weeks of gestation, weighing 200 g or less, and infants with congenital anomalies	Time-series regression	*N* = 482,765	- 10% (95% confidence interval 7%–14%) increase in risk of being born preterm in winter when compared with summer - no increased risk associated with exposure on the day of birth to daily mean levels of maximum and minimum temperatures, relative humidity, precipitation, hours of daily sunshine, mean barometric pressure or the largest daily drop in barometric pressure	Adjusting for public holidays, seasonality, day of the week and between year variations Daily mean levels of ambient ozone and particulate matter with aerodynamic diameter <10 micrometers; on the day of birth, cumulative exposure up to 1 week
[67]	Heat Index (function of temperature and humidity)	Preterm birth (<37 gestational weeks) Preterm labor	New York, NY, USA (NT), 21 March 1993–20 March 1994	-weekly averaged heat-humidity indexes (2 summer and 2 winter weeks that showed the with highest and lowest heat-humidity index values for each season)	Retrospective hospital cohort study	Excluded twins, patients with cerclage, and deliveries induced prematurely for obstetrical complications	Exact trend test	*N* = 3,972	- The preterm labor rate increased from 1.23% to 3.0% for an increase of heat-humidity index from 25 to 79.5 degrees F, *p* < 0.002) - For all preterm births, similar but not statistically significant trend (*p* < 0.29)	[67]
[62]	Temperature	Preterm birth (extreme immaturity (<28 completed weeks of gestation) and preterm birth (28–36 completed weeks))	Brisbane, Australia (NT), 2005–2009	- weekly mean temperature and relative humidity in the 4 weeks or the week preceding the time at risk From week 15 of gestation to birth	Hospital cohort study	All births with conception dates ranging from 19 weeks before the cohort study to 43 weeks before it ended	Cox proportional hazards model with gestational age as the time axis	*N* = 101,870	The hazard ratio for a live preterm infant (28–36 weeks) was 1.20 at 27 °C as compared with the reference temperature of 21 °C. - Humidity adjusted for but no results reported.	Particulate matter with aerodynamic diameter <2.5 µm and <10 µm, ozone, nitrogen dioxide, carbon monoxide and sulfur dioxide; sex of the infant, maternal age, smoking, marital status, indigenous status, preeclampsia and gestational hypertension).
[77]	Seasonality of birth (month)	Preterm birth (<37 gestational weeks)	Greece (entire country) (NT), 1980-2008	Month of birth - winter (December–February) - summer (June–August), - fall (September–November) - spring (March–May).	Retrospective cohort study	live births at or beyond 24 gestational weeks	Time series Logistic regression	3,217,738 births 15,150 (4.71%) premature	Preterm births risk higher during the winter and summer months	Maternal age, sex
[73]	Barometric pressure	Labor onset	Massachusetts, USA (NT), October 1993–October 1994.	- Daily mean barometric pressure (lowest tercile compared with the other two terciles) 3 consecutive hours of falling or rising barometric pressure	Retrospective hospital cohort study	All women with spontaneous onset of labor between 37–42 weeks	Least squares regression comparing onsets of labor occurring in the lowest tercile of daily mean pressure compared to other terciles	*N* = 2,435	No significant difference in spontaneous onset risk between days with high or low pressure Significant decrease in the onset of labor after 3 consecutive hours of falling, but not rising, pressure	Parity, date and time of onset of contractions, date and time of spontaneous rupture of membranes, date and time of delivery, sex of infant, type of delivery
[72]	Barometric pressure	Labor onset Premature rupture of membranes	Tokyo, Japan (NT), January 1997–December 2003	- Averaged (daily?) barometric pressure assigned women to two groups (>1010.7 hPa or <1010.7 hPa at delivery)	Retrospective hospital cohort study	All infants who had a spontaneous cephalic delivery in this hospital	Paired *t* test or Wilcoxon’s signed rank test	*N* = 2,278	- No significant association between onset of labor and barometric pressure. - Increase in the number of rupture of the membranes at lower than 1,010.7 hPa (*p* < 0.01). - Increase in the number of deliveries on days with larger changes in barometric pressure (decreasing or increasing), (*p* < 0.01).	Membrane rupture, maternal age, fetal gender, parity, birth weight, gestational age
[74]	Barometric pressure	Labor onset	Texas, USA (NT), 1992	- rapid decrease in barometric pressure defined as drop of 0.06 inches of mercury in 24 h	Retrospective descriptive study	All births 36 weeks of gestation or more, spontaneous onset of labor, delivered at hospital during 24 h before/after pressure drop	Chi square test	*N* = 162	- Significantly more occurrences of labor after drop in barometric pressure than before (*p* = 0.02)	None

^a^ NT: Non-tropical climate; T: Tropical climate.

### 3.3. Findings from Birth Weight

We identified 27 studies that examined different forms of birth weight as outcomes (Table 3): mean birth weight as a continuous variable (in term births (*n* = 7) or in all births (*n* = 14)), low birth weight (LBW < 2,500 g) (in term births (*n* = 2) or in all births (*n* = 6)), and small for gestational age (SGA, *n* = 4).

Four studies focused on seasonality of conception and all were conducted in non-tropical settings, but could not be pooled since they covered different outcomes. A study of 3,333 births in Turkey associated conception in summer and autumn with lower mean birth weights in term born infants [78]. A study of 291,517 births in Germany reported an increased risk of term LBW for conceptions in spring [66]. A study of 188,276 births in the USA associated conception in winter and spring with an increased risk of SGA or LBW [75]. Another USA study of 1,435,213 births associated conceptions in summer months with higher mean birth weights (whether gestational age was adjusted for or not), and conception in spring was also associated with a slight trough in mean birth weight [50].

**Figure 5:**
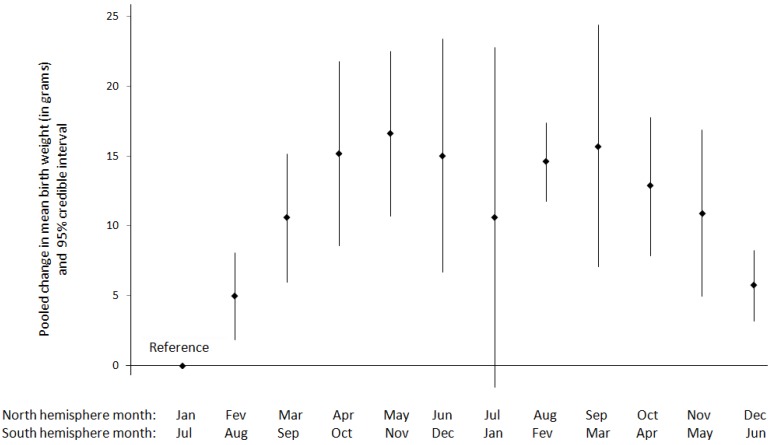
Pooled change in mean birth weight (and 95% credible interval) by month of birth in term born infants (*N* = 5,398,360 births).

**Figure 6:**
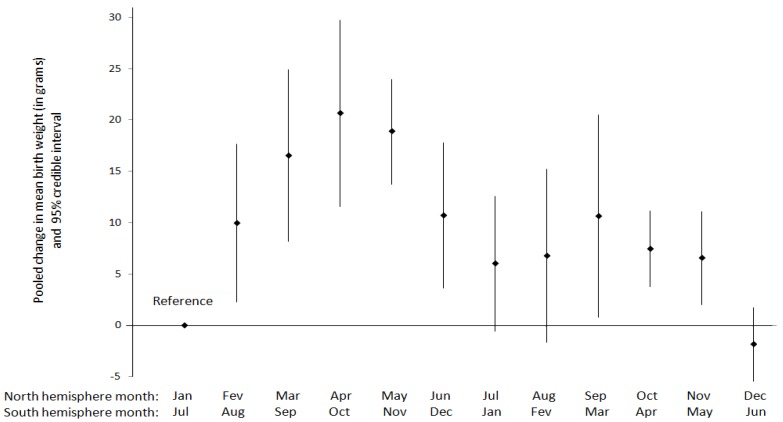
Pooled change in mean birth weight (and 95% credible interval) by month of birth in all infants (*N* = 70,652,872 births).

Thirteen studies examined mean birth weight and the time of birth (Table A20). Three focused on month-to-month variations in term born infants [79,80,81] and six on term and preterm births combined (Table A20) [53,56,82,83,84,85]. Meta-analyses were conducted separately for these two outcomes and included 5,398,360 and 70,652,872 births, respectively. The temporal patterns observed for each of these outcomes appear similar Figure 5, Figure 6: the lowest birth weights are observed during the coolest months of birth (December/June and January/July for the North/South hemisphere respectively), rise in the spring, slightly drop during the summer with a trough in July, and rise again in autumn.

Four articles analyzed the variations in mean birth weight only by season of birth. No meta-analysis was conducted because of insufficient papers for specific outcomes. In the Mediterranean setting of Greece a study of 516,874 births found mean birth weights to be lower during spring and summer than during other seasons [54]. Other studies reported mixed findings but all together included a lower number of 49,399 births [86,87].

Three studies focused on infants born SGA by season of birth. In a non tropical setting in Australia, a study of 147,357 births reported no difference in the odds of SGA between seasons [88]. Two tropical studies, in Indonesia [32] and the Gambia [58] found higher risks of SGA for infants born during the rainy season compared to the dry season.

Three studies examined term LBW by season of birth. In Germany, term LBW was associated with birth in winter [66]. Two tropical studies in Indonesia [32] and Tanzania [89] associated term LBW with birth in the rainy season.

Four studies focused on LBW (in term and preterm infants combined) by season of birth. In the entire USA, LBW risk was highest in the winter, and second highest in the summer [56]. In Greece, the highest LBW risk was observed in the summer [54] whereas in Israel no association was observed [83]. One study conducted in a tropical setting in Australia reported a significantly higher LBW risk for infants born during the wet than during the dry season [85].

The effects of temperature on birth weight were assessed in thirteen studies, with different questions explored by several approaches: some studies focused on the association between birth weight and the climate (or “temperature regime” [90]) prevailing in different locations, that was typically reflected by mean annual temperature [90,91,92]. These analyses were based on geographical contrasts in annual mean birth weights between locations of different climates. Other studies focused on temporal contrasts in exposure in fixed settings, with a focus either on the temporal contrasts in temperature typically experienced between different trimesters of pregnancy [80], or on the occurrence of extreme climatic events [92,93].

A pooled analysis of 140 populations from countries spanning all continents examined the relationship between mean birth weight and climates using a heat stress index defined as a combination of daily maximum temperature and afternoon humidity, subsequently averaged by year [91]. This analysis reported an inverse association between heat stress and mean birth weight, after controlling for covariates (altitude, latitude, mortality index, energy intake, gross domestic product and maternal height). This work was recently extended [90]; it was estimated that under projected climate change, mean birth weight will decrease by 0.44%–1.05% per °C increase in temperature. A similar analysis was conducted in the USA and reported an inverse association between annual average temperature of and mean birth weight at the county resolution [92].

Three studies examined the average temperature exposure by pregnancy trimester and mean birth weight in term born infants, but they could not be pooled since one of them used a dichotomous indicator for temperature [94]. A study of 418,817 births in Ireland reported a 3.5 g increase in mean birth weight in females and 1 g in males per 1 °C increase in the mean daily maximum temperature during the second trimester only [80]. A smaller study in Turkey (3,333 births) reported an association of similar magnitude, also for the second trimester [78]. However, a study of 8,516 births in New Zealand reported no effect of temperature “peaks” and “troughs” during any trimester on birth weight [94].

Three other studies assessed similar associations but did not exclude preterm births. Again, no meta-analysis was conducted because of differences in temporal resolutions of temperature indicators. A study of 516,874 births in Greece reported an inverse association between mean birth weight and the mean temperature during the month of birth [54]. Another study of 225,545 births in Israel found positive associations between birth weights and mean daily maximum temperature in the first pregnancy trimester [83]. On the contrary, a study of 12,150 births in Scotland reported inverse associations between birth weight and mean ambient temperature in the mid 10-day period of the first trimester and no association for the second trimester. However a positive association was observed for the third trimester [82].

Three studies focused on the associations between temperature and categorical birth weight indicators. In Australia, an increase in average temperature during the entire pregnancy was associated with increased odds of SGA [88]. In Germany, no association was observed between term LBW (<2,500 g) and temperature in any trimesters of pregnancy [66]. However in Sweden, very low birth weight (<1,500 g) was associated with colder than expected temperatures during summer months [95].

Two studies specifically examined the impact of extreme temperature episodes on mean birth weight. An analysis in the entire USA showed that the higher the number of days with temperature exceeding >85 °F within each pregnancy trimester, the lower the mean birth weight [93]. The number of days with temperature < 25° F during the first trimester was also associated with a decrement in mean birth weight, suggesting a possible inverse U-shaped relationship. A subsequent analysis that explored even more extreme events (days <20 °F and >90 °F) confirmed such relationships [92].

In the five studies examining sunlight hours or daylight hours (Table 3), findings were mixed across non-tropical settings. Studies in Ireland [80] and Turkey found no association between term birth weight and sunlight for any trimester of pregnancy [78]. Another study in Australia [88] found no association between sunlight and SGA. However, two New Zealand studies found that mean birth weight was positively associated with mean sunlight hours during the first trimester of pregnancy and inversely associated with sunlight hours during the second and third trimesters [94,96]. No study reported any association between rainfall (mm) during pregnancy and birth weight [78,80,83].

**Table 3:** Associations between meteorology and birth weight.

Reference	Seasonal or Meteorological Variable	Outcome	Setting (climate type ^a^), study period	Exposure metric	Study Design	Inclusion criteria	Statistical method	Population size	Summarized Main Results	Confounders adjusted for/other comments
[81]	Season of birth (month)	Term birth weight (continuous variable)	Warsaw, Poland (NT), May 2004-April 2005	- Year divided into four seasons: Spring (April–June); Summer (July–September); Autumn (October–December) and Winter (January-March)	Hospital based cohort study	All singleton live births after 36 weeks of pregnancy	One way analysis of variance of birth weight transformed to z score Weighted Spearman rank correlation	*N* = 10,631	- Average Z-scores for birth weight associated with month of birth for boys (*p* = 0.01) respectively, and for girls (*p* < 0.01). - Peak Z-score values for boys born in October with a trough in March. Peak Z-score values for girls born in July and August with a trough in April. -No association between birth weight and season of birth.	None
[78]	Seasonality of conception (month) Temperature Humidity Rainfall Daylight	Term birth weight (continuous variable)	Istanbul, Turkey (NT), 1992–2003	- Women were divided into four groups according to season of last monthly period - Year divided into four seasons: Spring, Summer, Autumn and Winter - Mean daily temperature (°C) and humidity (%), total daily rainfall (mm) and daily duration of daylight (hours) for each trimester of pregnancy	Hospital based cohort study	All live births after 36 weeks of gestation, except multiple pregnancies	Stepwise multiple linear regression	*N* = 3,333	- Women who conceived in winter and spring were exposed to higher temperatures during the second trimester and delivered babies with higher birth weights than those who conceived in summer and autumn. Regression parameter for “Temperature to which the subject was exposed during the second trimester (°C)”: 0.001 multiples of the mean. The mean being about 3,700 g, The gain would be about 3.7 g per °C.) - No association between birth weight and humidity, rainfall, and daylight in any trimester.	maternal age and parity, mode of delivery, sex
[80]	Seasonality of birth (month) Rainfall Sunshine Temperature	Term birth weight (continuous variable)	Northern, Ireland (NT), 1971–1986	- Year divided monthly -Mean daily maximum and minimum temperatures, rainfall, and hours of bright sunshine for each pregnancy trimester	Population based cohort study	Singleton live births after 36 completed wks of gestation	Linear regression	*N* = 418,817	- The lowest adjusted mean birth weights were 25.5 g, 29.6 g, and 31.6 g lower in May, June, and July, respectively, than in January - In females, an increase of 1 °C in the mean daily maximum temperature during the second trimester was associated with an increase in mean birth weight of 3.5 g. (SE 0.88) -In males 1.02 (SE 0.88) -No significant association for other trimesters or for rainfall, sunshine, or mean daily minimum temperature	-Year of birth, duration of gestation, maternal age, number of previous pregnancies, sex, and social class
[89]	Seasonality of birth (rainy *vs*. dry)	Term birth weight (continuous variable) Term low birth weight (<2,500 g)	Morogoro, Tanzania (T),	N/A	Hospital based cohort study	All live singleton babies at full term gestation	N/A	*N* = 19,783, including 2,354 low birth weight infants	- Mean birth weight low during the rainy season and high during the dry season - Low birth weight incidence higher during the rainy than the dry season	Food intake, energy expenditure
[87]	Seasonality of birth (4 seasons)	Term birth weight (continuous variable)	12 cities in the USA (NT) 1959–1965	- Year divided into four seasons: winter (December, January, February); spring (March, April, May); summer (June, July, August); and fall (September, October, November)	Multi- hospital based cohort study	All live births at full term gestation	ANOVA Multiple linear regression	*N* = 24,325	Infants born in fall had lower birth weight than those born in winter (*t* test = 2.15, *p =* 0.03) and spring (*t* test = 2.48, *p =* 0.01), but no association remained after adjustment for confounders.	Sex, race, maternal age, maternal education, maternal BMI, first born, ever breast fed, weight gain in first four months
[79]	Seasonality of birth (month)	Term birth weight (continuous variable)	Chile (entire country) (NT), 1987–2007	Year divided monthly and regionally: North, Central-coast, Central-interior and South	Population based cohort study	All live-born singletons with gestations between 37 and 41 weeks in study period	Multivariate regression	*N* = 4,968,912	Birth weight has a bimodal peak in spring (*p* < 0.001) and fall (*p* < 0.001) and a pronounced nadir in winter and smaller nadir in summer	Maternal age, marital status, college education, urban region
[86]	Seasonality of birth (4 seasons)	Birth weight (continuous variable)	Rome, Italy and Sassary, Italy (NT), January 1993–December 1996	- Year divided into four seasons: winter, spring, summer, autumn	Hospital based cohort study	All live births	Variance analysis	*N* = 5,291	- Birth weight is significantly lower in infants born in winter than in autumn. - mean differenceis 327 g (*p <* 0*.*003); after correction for multiple comparison (*p <* 0*.*02)	Population and gestational duration, season of birth (with birth weight as dependent variable)
[54]	Seasonality of birth (4 seasons) Temperature	Birth weight (continuous variable) Low Birth Weight (<2,500 g)	Greece (entire country) (NT), 1999–2003	- Year divided into four seasons: winter (December–February), spring (March–May), summer (June–August), and autumn (September–November) - mean air temperature during month of birth	Population based cohort study	All Greek citizens born or deceased during the period of study	Chi square Tests for contrasts in low birth weight probability between seasons Pearson’s R for association of continuous birth weight with temperature	*N* = 516,874 born *N* = 554,101 died	- Infants born during autumn and winter had higher birth weight than those born in other seasons of the year - Low birth weight rates were lower (*p* < 0.05) for infants born during the autumn and winter seasons. -Mean air temperature during the month of birth associated with birth weight r = −0.218 (*p* < 0.001)	None
[91]	Temperature regime (climate, not seasonal variation) reflected by heat stress (humidity and temperature)	Birth weight (continuous variable)	140 populations from the WHO (1992) population data	Heat stress index considering yearly average of maximum daily temperature and afternoon humidity	Pooled analysis of population based studies	Population with specific data on birth weight and thermal climate.	Linear regression	140 populations	- Significant correlation between heat stress and birth weight R^2^ = −0.59 (*p* < 0.001)	Data on both birth weight and heat stress were reduced to an annual average value, thus ignored seasonal variation in climate
[90]	Temperature regime (climate, not seasonal variation)	Birth weight (continuous variable, log-transformed)	63 countries from the WHO (1992) population data 1971–2000	Climate characterized by the mean of daily minimum and maximum temperature from the coolest and warmest months, respectively	Ecological study		Linear regression	63 countries, number of births not provided	Overall reductions in BW at increasing mean temperatures vary from 0.44% per °C in temperature range 0–5 °C to 1.05% per °C in the temperature range 20–25 °C, subject to adjustment for variation in nutrition, altitude and age of motherhood.	Altitude, prevalence of under-nourishment, obesity, mean age at motherhood, fertility rate, malaria prevalence, geographic origin.
[93]	Extreme temperatures	Birth weight (continuous variable)	USA (entire country), 1972–1988	Number of days within each pregnancy trimester that fall into different bins of daily average temperature (average of maximum and minimum temperature) <25° F, 25°–45° F, 45°–65° F, 65°–85° F, >85° F). Aggregated at the county level	Cohort study	Mothers aged 16–45, 48 continental states + DC	Linear regression	*N* = 37,100,000	As compared to 45–65° F, each additional day <25° F is associated with a −0.000025 (95% CI: −0.00001; −0.00004) detriment in log birth weight each additional day>85° F is associated with a −0.000025 (95% CI: −0; −0.00005) detriment in log birth weight Linear relationships for temperature exposure during the 2nd and 3rd trimesters: <25° F is associated with a 0.000025 (95% CI: 0.00001; 0.00004) increment in log birth weight each additional day> 85° F is associated with a −0.000075 (95% CI: −0.00006; −0.000012) detriment in log birth weight	Smooth function for the date of conception. Conditioning by county and year, mother’ s age, fertility history, educational level and marital status Inverse U-shaped dose response relationship between log birth weight and number of days falling within the different bins during the first trimester
[92]	Temperature regime (climate) Extreme temperatures	Birth weight (continuous variable)	USA (entire country) (NT), 1974–1978 And 1984–1988	Number of days during the month or season of birth that fall into different categories of daily average temperature: <20° F,<25° F, 25°–45° F, 45°–65° F, 65°–85° F, >85° F, 90° F, >95° F Winter (December to February), Spring (March to May), Summer (June to August), Fall (September to November) -study period+ annual average temperature from 1960 to 1969 (“climate”)	Ecological study (county-level resolution)	20% sample of White mothers aged 19 to 38	Multilevel linear regression with spatial autocorrelation terms	4,921,561	The warmer the yearly average temperature of a county, the lower the birth weight. After controlling for these climatic patterns, birth weight was inversely related to both extremely cold and extremely hot temperatures. In birth month (1974–1978): birth reduction associated with each day *<*20° F: −0*.*0761 (SD: 0*.*0734) *>*90° F: −0*.*7449 (SD: 0*.*0802) With mean county temperature: −1*.*1409 (SD: 0*.*3683) In birth month (1984−1988): birth reduction associated with each day *<*20° F: −0*.*4749 (SD: 0*.*0739) *>*90° F: −0*.*2927 (SD: 0*.*06147) With mean county temperature: −4*.*7054 (SD: 0*.*2594)	County per capita income, average elevation
[53]	Seasonality of birth (month)	Birth weight (continuous variable)	Japan (entire country) (NT), January 1974–December 1983	Year divided into spring (March–May), summer (June–August), autumn (September–November), and winter (December–February)	Time series analysis	All live singletons	Time series regression	*N* = 16,796,415	Significant inter-seasonal variability in mean birth weight (*p* < 0.001): two peaks in May and October–November and two troughs in June–September and December.	None
[50]	Seasonality of conception (month)	Birth weight (continuous variable) Adjusted or not for gestational age	NJ (entire state), USA, (1997-2006), New York, NY, USA, (1994–2004) PA (entire state), USA, (2004–2010)	Month of conception	Retrospective cohort study	Single births with no missing information on gestation length	Cohort study based on comparison between siblings	*N* = 1,435,213	Gain of 8–9 additional g for summer conceptions compared with January conceptions (both before and after adjusting for gestational age)	Stable maternal characteristics (by design) Influenza Gestational age
[82]	Seasonality of birth (Month) Temperature	Birth weight (continuous variable)	Aberdeen, Scotland (NT), 1950–1956	-Year divided into Winter (December–February); Spring (March–May); Summer (June–August and Autumn (September–November) -Mean ambient minimum and maximum temperature for 10 days around conception, the middle of Each pregnancy trimester	Population based cohort study	All births	Linear regression models	*N* = 12,150	-lowest birth weights in the winter months (December–February) and highest in the autumn months (September–November) -1 °C increase in mean ambient outdoor temperature in the mid 10-day period of the first trimester -first trimester associated with a 5.4 g (95% CI 2.9, 7.9 g) decrease in birth weight -second trimester associated with a 1.8 g (95% CI −0.7, 4.3 g) decrease in birth weight -third trimester associated with a 1.3 g (95% CI 0.50, 2.1 g) increase in birth weight	Sex, maternal age, birth year, birth order, social class
[96]	Sunlight	Birth weight (continuous variable)	Dunedin, New Zealand (NT), August 1967–July 1978	-Daily sunlight maximal hours during pregnancy	Hospital based cohort study	All singleton live births	Cross-correlation functions from Fourier transforms	*N* = 20,021	- Monthly means for neonate weight varied sinusoidally with monthly variation in mean bright sunlight hours - effect of mean sunlight hours on birth weight most evident when maximal sunlight was positive during the first 3 pre-natal months and negative during the last 6 pre-natal months.	None
[84]	Seasonality of birth (4 seasons)	Birth weight (continuous variable)	Queensland, Australia (T), January 1987–December 1999	Year divided into spring (September–November); summer(December–February); fall (March–May); winter(June–August)	Time series analysis	All singleton pregnancies with a gestation of at least 37 weeks	Spectral analysis	*N* = 350,171	Winter and spring infants born slightly heavier compared to summer and autumn born infants (25-g difference between neonates born in October *vs*. May).	None
[94]	Temperature Sunlight	Term Birth weight (continuous variable)	Dunedin, New Zealand (NT), January 1999–December 2003	Temperature and sunshine hours by pregnancy trimester	Hospital based cohort study	Full term births >38 weeks of gestation	One-way analyses of variance	*N* = 8,516	- No association between birth weight and temperature in the second trimester. -Infants exposed to high levels of sunshine during the first trimester born heavier than infants exposed to low levels of sunshine. - Infants whose mothers were exposed to trough periods of sunshine during their second and third trimesters heavier than infants whose mothers who were exposed to peak periods of sunshine during the same trimesters	None
[56]	Seasonality of birth (month)	Birth weight (continuous variable) Low birth weight (<2,500 g)	USA (entire country) (NT), 1989–2001	Year divided into months	Population-based cohort study	All birth certificates	Linear regression	52,041,052	Children born in December and January have lower average birth weights than other children Infants born in April weigh 23.3 grams more on average than those born in January Early spring and late summer births are less likely to have a low birth weight	
[83]	Seasonality of birth (month) Temperature Precipitation	Birth weight (continuous variable) Low birth weight (<2,500 g) High birth weight (macrosomia >4,000 g)	Israel (entire country) (NT), 1998–2004	-Year divided into seasons: winter (December–February), spring (March–May), summer (June–August), and fall (September–November) - monthly means of maximum and minimum daily temperature, precipitation, and number of rainy days	Population based cohort study	All live births	Linear regression (mean birth weight) Logistic regression (Low birth weight)	*N* = 225,545	- Significant association between birth weights and season with a peak in July and trough in January - No association between low birth weight and seasonality -Babies born in summer had an OR = 1.12, 95% CI (1.07–1.18) for macrosomia compared with winter. - Positive association between mean birth weight and monthly minimal temperatures at the first month of first and third trimesters. - Monthly means of precipitation and number of rainy days not associated with birth weight	Maternal age, sex, year of birth, maternal diabetes
[85]	Seasonality of birth (month)	Birth weight (continuous variable) Low birth weight (<2,500 g) Very low birth weight (<1,500 g)	Kimberly, Australia (T), 1981–1993	Year divided into seasons: very hot summer (January–June) and heavy rainfall from (January–April); Winter (July–December)	Population based cohort study	All singleton live births	Logistic regression analysis: OR of wet season compared to dry season	*N* = 4,058	- Mean birth weight varied by month of birth (*p* = 0.003) -low birth weight more common during the wet season: OR 2.73; 95% CI (2.3–3.67) with the lowest birth weight in March - Increased risk of very low birth weight during the wet season compared with the dry season: OR 2.73; 95% CI (2.3–3.67), but low birth weight not associated with the wet season OR 1.06; 95% CI (0.96–1.17; *p* = ns)	None
[75]	Seasonality of conception (4 seasons)	Low birth weight (<2500 g) Small for gestational age (<10th percentile of birth weight for gestational age)	NC (entire state), USA (NT), 2001–2005	-Season defined as: winter (December–February), spring (March–May), summer (June–August), and fall (September–November)	Population based cohort study	Singleton first births to non-Hispanic white and black women, excluding births with missing covariate data, congenital anomalies, birth weight <400 g, extremely high or low gestational age, and maternal age >44 years	Linear regression for mean birth weight logistic regression for low birth weight and small for gestational age	*N* = 188,276	-Spring and winter conceptions were associated with higher rates of low birth weight for gestational age among statewide births (*p* < 0.05), as well as among rural county births for the non-Hispanic white group (*p* < 0.05). - Rates of small for gestational age were lowest among non-Hispanic white group spring conceptions across all North Carolina counties, urban, and rural counties (*p* < 0.05)	Maternal age, education level, marital status, smoking status, region of North Carolina, county urbanization
[58]	Seasonality of birth	Small for gestational age (<10th percentile of reference standard gestational age)	Keneba, Manduar, and Kantong Kunda, The Gambia (T), (3 villages of the West Kiang District) 1976–2003	Year divided into agricultural season that revolves around the rainy season (July–November).	Population based cohort study	All live births	Fourier series	*N* = 1,916	Incidence of SGA highest at the end of the annual hungry season, from August to December (peaking in November at 30.6%), with a nadir of 12.9% in June.	Thick and thin blood smears obtained from antenatal clinics to measure malarial infection; activity diaries and 24-hour activity recall to assess maternal workload
[66]	Seasonality of conception and birth (4 seasons) Temperature	Term low birth weight (<2,500 g)	Brandenburg, Germany (NT), 2002–2010 Saxony, Germany (NT), 2005–2009	-Year divided into four seasons: December to February (winter), March to May: (spring), June to August: (summer), September to November: (autumn) -Daily mean temperature for each trimester of pregnancy	Time series analysis	All singleton births ≥37 weeks of gestation and with birth weight greater than 200 g	Logistic time series regression Fourier series	Brandenburg *N* = 128,604, including 6,242 low birth weight infants Saxony *N* = 162,913, including 8,034 low birth weight infants	- Association between low birth weight and conception in Spring in Brandenburg OR = 1.19, 95% CI (1.05–1.35) - Association between low birth weight and birth in Winter in Brandenburg OR = 1.15, 95% CI (1.02–1.30) - No association between low birth weight and temperature in Brandenburg in first OR = 0.93, 95% CI (0.70–1.23), second OR = 0.91, 95% CI (0.66–2.25), or third trimester OR = 0.86, 95% CI (0.64–1.17) - No association between low birth weight and temperature in Saxony in first OR = 0.89, 95% CI (0.70–1.12), second OR = 1.09, 95% CI (0.82–1.45), or third trimester OR = 1.15, 95% CI (0.87–1.52)	Maternal age available for Saxony only
[95]	Temperature	Very low birth weight (<1,500 g)	Sweden (entire country), (NT), 1973–2010	Mean daily temperature averaged for the month of birth	Population based cohort study	All singleton live births during the summer season (June, July, August)	Time series analysis	*N* = 3,757,440	- Inverse association between very low birth weight risk and mean monthly temperature in summer season - 13.6% increase in odds of a very low birth weight male for a colder than expected June and 5.4% increase in odds for a colder than expected August	
[32]	Seasonality of birth (rainy *vs*. dry)	Low birth weight (<2,500 g) Small-for-gestation age (birth weight <10th centile of the gestational age- and sex-specific US reference for fetal growth)	Lombok, Indonesia (T), 2001–2004	Year divided into rainy season (November–March) and dry season (April–October)	Double blind cluster randomized controlled trial	All singleton live births with birth weight measured within 72 h of birth	Hierarchical logistic regression	*N* = 14,040	22% increased odds of low birth weight in babies born in the rainy season; 18% increased odds of small for gestational age in babies born in the rainy season	Infant’s sex, season at birth, mothers’ residence, nutritional status, education, household wealth, mid-upper arm circumference, height and a composite variable of birth order and pregnancy interval
[88]	Seasonality of birth (4 seasons) Temperature Sunlight	Small for gestational age and sex (infants with a weight for gestational age <10th percentile for their sex) Proportion of optimal birth weight (POBW)	Perth, Australia (NT), 1998–2006	-Year divided into seasons: winter (June–August) and summer (December–February) -temperature and sunlight averaged over the entire duration pregnancy and over each trimester of pregnancy separately.	Population based cohort study	All singleton live births ≥400 g birth weight and/or ≥20 weeks’ gestation	Multiple linear regression, multivariate models	*N* = 14,7357	- POBW with third trimesters predominantly in summer was 0.18%, 95% CI (0.00%–0.36%) lower than for those in winter. - No association between season of birth and small for gestational age - Inter-quartile range increase in temperature during entire pregnancy (0.73 °C) was associated with small for gestational age and sex with an OR = 1.02, 95% CI (1.00–1.05). - POBW decreased by 0.14%, 95% CI (0.01%–0.27%) per inter-quartile range increase in third-trimester temperature (9.15 °C). - No significant effect observed for sunlight exposure	Criteria air pollutants: Particulate matter with aerodynamic diameter <2.5 micrometers and <10 micrometers, ozone, nitric oxide, nitrogen dioxide and carbon monoxide

^a^ NT: Non-tropical climate; T: Tropical climate.

## 4. Discussion

The results of this systematic literature review show that preeclampsia, eclampsia, gestational length and birth weight are seasonally patterned. The risks of preeclampsia appear higher for women with conception during the hottest months, and delivery in the coldest months of the year. Delivery in the coldest months is also associated with a higher eclampsia risk. However, direct evidence of the effects of temperature on preeclampsia and eclampsia is still insufficient. Patterns of decreased gestational lengths have been observed for births in winter, as well as summer months. Several recent studies also report decreases in gestational lengths associated with high temperature during the month of, or the few days preceding, delivery. Birth weights (either in all or in term born infants) are lower for deliveries occurring in winter and in summer. Only a few studies investigated the relationships between birth weight and temperature or sunshine exposure, which does not allow drawing conclusions on these relationships.

We identified several seasonal patterns of pregnancy outcomes after synthesizing available evidence via a meta-analysis approach. Overall, more studies documented variations in the risk of pregnancy outcomes related to the time of birth than the time of conception. The absence of individual information on gestational length in available papers precludes any rigorous comparison of results from studies based on the season of conception and the season of birth. We therefore examined these two exposure times separately. Most studies, and importantly, the biggest studies that carry more weight in the meta-analysis, have been conducted in non-tropical countries. We acknowledge that the patterns described above are mostly representative of non-tropical countries.

The patterns of higher preeclampsia risks for women with conception during the hottest months, and delivery in the coldest months might be explained by some direct effects of exposure to heat during the first trimester of pregnancy, and to cold temperatures at the end of pregnancy, both of which are biologically plausible [18,21]. However, available studies on the associations between measured temperature and preeclampsia do not provide sufficient evidence to draw conclusions on these relationships and further studies would be needed to explore them further.

The bimodal seasonal patterns observed for lower lengths of gestation and birth weights in winter and summer also call for explanations. Some researchers pointed at different seasonal patterns of time of conception correlated with sociodemographic profiles (e.g., age, education level, race/ethnicity, marital status) [56,97]. It was hypothesized that these differences in seasonal patterns of time of conception across socio-demographic groups might explain seasonal patterns in adverse pregnancy outcomes, since mothers with different sociodemographic characteristics experience contrasted risks of adverse pregnancy outcomes [97]. This hypothesis was recently examined in the USA by a sibling study that controlled for maternal characteristics by design [50]. This study concluded that seasonality of conception due to sociodemographic profiles might contribute no more than 22% of variation in gestational length by season.

Some large studies suggest potential influences of temperature extremes on the observed seasonal patterns in gestational length and birth weight [61,92,93]. In Japan, peaks of preterm births were identified both in winter and summer, but the winter peak was most prominent in the North of Japan (that experiences a cooler climate), whereas the summer peak was most prominent in the South of Japan (that experiences a hotter climate) [61]. The observation of short-term associations (lag time of a few days) between heat and reduced length of gestation [59,63,68,69] also provide convincing evidence, especially since associations on such short temporal scales cannot be explained by socioeconomically differentiated seasonal patterns in the time of conception [97]. Last, a large study in the USA reported reductions in birth weight associated with the number of extremely hot or cold days during the month or the season of birth [92]. If such associations were causal and reflected the effects of extreme temperatures on birth weight, temperature might contribute to explain the bimodal seasonal pattern we observed for birth weight (*i.e.*, a trough during the summer and winter).

These observations justify exploring further the association between specific meteorological parameters and pregnancy outcomes. However, meta-analysis could not be applied to summarize available evidence on these relations (with the exception of preeclampsia and mean temperature during the month of birth for which a pooled correlation coefficient could be estimated) because the definitions of exposure metrics for meteorological parameters varied substantially between studies, even when a single pregnancy outcome was considered. These exposure metrics differed in nature (e.g., heat waves with different definitions [68], maximum or minimum daily temperature as a continuous variable [69], indices combining temperature and humidity [59]). Some composite indicators were calculated out of the same variables (e.g., temperature and humidity) but using different formulae (apparent temperature [69], heat-humidity indices [67]). Indicators used to study the same outcome were frequently of different temporal resolutions (pregnancy trimester, month/week of birth, month, or a few days preceding birth). In addition, some authors conducted analyses using categorized exposure metrics defined according to different thresholds for meteorological parameters [94].

The diversity of the natures, temporal resolutions and categorizations used to defined exposure to meteorological variables have advantages in representing a wealth of hypotheses (e.g., related to the respective effects of acclimation, of exposures averaged on specific periods of fetal development and of sudden changes toward temperature extremes). However, these differences also hamper conducting a rigorous meta-analysis to synthesize such diverse information. Even if more studies were available, important differences between definitions of meteorological variables and other methodological aspects would likely persist, unless a more coordinated research strategy is defined. Multi-centric studies using harmonized methodologies, (as was done recently for air pollution and pregnancy outcomes [98]) would better help address current research needs. Since routine (*i.e.*, daily, hourly) meteorological parameters are collected throughout the world, such coordination appears technically feasible. Centralizing such meteorological data from different regions (along with the corresponding data for pregnancy outcomes) would then allow for a posteriori calculations of specific (either previously used or new) exposure metrics across regions and exploring hypotheses within a consistent and powerful framework. Especially, the exploration of non linear relationships between temperature and pregnancy outcomes could more effectively be explored via such a pooled analysis than via a meta-analysis (relying either on a single parameter from regression models assuming linear dose-response relationships, or on categorized indicators based on various cut-points defined by uncoordinated investigators).

Beside the above sources of heterogeneity, most published studies share a number of methodological limitations. Only a few of them explored the influence of some established or potential risk factors for pregnancy outcomes that follow seasonal patterns and/or are correlated with meteorological conditions [99]. Such factors might act as confounders in the observed associations between meteorological variables and pregnancy outcomes. Among them stands ambient air pollution which is strongly influenced by meteorology. Only six studies included in our review adjusted for air pollution, though no evidence for confounding was reported in any of these studies. Several studies reported positive associations between temperature and preterm births [62,63,68,69] or birth weight [88] even after adjusting for air pollutants, whereas another study on preterm birth reported no association [65].

Infection is an established risk factor for preeclampsia [100], preterm birth and low birth weight [101], although the full array of infectious agents leading to these outcomes is probably not known. Malaria may induce preterm delivery and low birth weight [101] and influenza has been suspected to cause preterm birth [50]. Many infections follow seasonal patterns and are influenced by meteorological factors including malaria, influenza [8] and genital tract infections [14,70]. For instance, a high humidity may increase micro-organisms proliferation, thus increasing the odds of infection. Rainfall, a direct cause of humidity, contributes to the spread of several infectious diseases [8]. Infections might therefore either mediate or confound the association between seasonality and/or meteorological conditions and pregnancy outcomes [50,69]. However, only a few studies accounted for maternal infection in this review, either by statistical adjustment or via applying exclusion criteria [50,52,58,59,69,71,102].

Maternal nutrition is another factor potentially correlated with seasonality and/or meteorology and pregnancy outcomes. Nutritional status encompasses a range of factors that vary seasonally, including availability of vegetables and fruits [82] and dietary intake [31]. Such factors may exert a direct effect on birth weight. Only a few studies measured maternal nutrition [32,89,90] and only one adjusted for it [90].

Ecological estimates were used to estimate exposure to meteorological factors in all studies. No study utilized individual modeling of exposure, and none considered the influence of time-activity patterns, heating, air conditioning and ventilation that may mitigate exposure of pregnant women to meteorological conditions.

Only a few studies employed statistical approaches dealing with temporal or spatial autocorrelation in data (e.g., [61,65,92]. Autocorrelation, if not accounted for, may notably result in erroneous variance estimates, and subsequent conclusions on the statistical significance of associations. Since both meteorological factors and pregnancy outcomes tend to be auto-correlated in space and time, the use of statistical approaches addressing these properties should be recommended for future studies.

Only one third of studies on birth weight or low birth weight focused on term born infants, and four on small for gestational age [32,58,75,88]. Only such studies taking the length of gestation into account (either by selecting term births only, or by using definitions of small for gestational age) allow examining the possible influence of meteorology on IUGR. Birth weight studies without consideration for gestational age do not allow disentangling whether any association between birth weight and meteorological factors is mediated by the influence of meteorology on IUGR and/or on gestational length. Clearly, more studies on term birth weight and small for gestational age are needed to address these issues.

An additional difficulty associated with research on meteorological factors is that different meteorological parameters tend to be correlated with each other: for instance, high barometric pressure (anticyclonic conditions) is associated with sunshine exposure, dry weather, high temperatures and low wind speeds. This may make the respective influence of each of these factors difficult to disentangle in individualized study settings. Yet, the diversity of climatic types on Earth offers a wide variety of combinations of meteorological factors. International multi-centric studies using harmonized methodologies would thus show improved potential to address these issues.

Several mechanisms have been proposed for the potential direct impact of meteorology on pregnancy outcomes. The hypertensive disorders of pregnancy such as preeclampsia have been hypothesized to be influenced by physiological responses to cold including vasospasm and ischemia [21]. Increases in blood pressure have been associated with cold temperatures in pregnant women [43]. Humidity might intensify cold-induced adrenergic discharges from cutaneous receptors [29]. Temperature and humidity effects on placental vascular development and spiral artery remodeling are also suspected [18]. Preeclampsia might also be related to seasonal variations of fluid balance, plasma volume, and osmolality [24], as well as sunlight effect possibly mediated by vitamin D levels [16,34].

Effects of heat stress on reduced length of gestation have been hypothesized [64,67], via heat-shock protein production [59], and dehydration, which could decrease uterine blood flow and increase pituitary secretion of antidiuretic hormone and oxytocin to induce labor [63]. Additional hypothesized pathways include temperature effect on blood viscosity and cholesterol levels [63] and a seasonal effect on maternal weight loss [58]. Barometric pressure might affect fetal hormone production, triggering preterm labor and preterm birth [72]. Last, experimental studies have shown that artificial changes in the light-dark cycle may induce onset of labor in rats [76]. For IUGR, proposed hypotheses include temperature effects on uteroplacental blood flow [80,84], changes in maternal energy expenditure [84,91] and sunlight effects on prenatal growth hormone production such as Vitamin D [94].

## 5. Conclusions and Recommendations

In conclusion, available research shows that the risks of preeclampsia appear higher for women with conception during the hottest months, and delivery in the coldest months of the year. Delivery in the coldest months is also associated with a higher eclampsia risk. However, direct evidence of the effects of temperature on preeclampsia and eclampsia is still insufficient. Patterns of decreased gestational lengths have been observed for births in winter, as well as summer months. Several recent studies also report decreases in gestational lengths associated with high temperature during the month of, or the few days preceding, delivery. Birth weights (either in all or in term born infants) are lower for deliveries occurring in winter and in summer. Only a few studies investigated the impact of temperature and sunshine exposure on birth weight, which does not allow drawing conclusions on these relationships.

Further etiological research is necessary to improve our understanding of the relationshipsbetween seasonality, specific meteorological parameters and adverse pregnancy outcomes. A few recommendations can be proposed to maximize the potential of future studies in the field:

- Further research should be preferentially conducted within the framework of international multicentric studies using harmonized methodologies. They would offer enhanced opportunities to disentangle the potential influence of different meteorological factors, thanks to the various combinations of these factors represented across Earth’s climates.
- Investigating non-linear relationships between meteorological parameters and pregnancy outcomes appears important.
- Future studies need to measure, and if necessary adjust for, risk factors that exhibit seasonal variability and may be correlated with meteorological factors such as nutritional patterns, air pollution and infections. Since nutritional pattern and maternal infections are seldom documented while meteorological stations are ubiquitous, research on the effects of meteorological conditions on pregnancy outcome might be most cost efficient if conducted within preexisting cohorts of nutrition and/or infections and pregnancy outcomes.
- They should ideally focus on individual indicators for exposure to meteorological conditions and cofactors, which would take into account time-activity patterns of pregnant women, and the mitigating effects of time spent indoors and associated heating, air conditioning and ventilation, on exposure.
- Future studies on birth weight should take into account the length of gestation as part of their study design, in order to disentangle the possible effects of meteorology on intrauterine growth restriction and/or the length of gestation.
- Lastly, fine temporal exposure windows over the entire gestational period are needed to identify critical windows of vulnerability to meteorological stressors.

Although such research efforts appear considerable, they would be worthwhile given the major impacts and high frequency of adverse pregnancy outcomes, and the seasonal patterns and suggested associations with meteorological parameters we identified in this review. Improved understanding would help proposing adequate recommendations for the prevention of adverse pregnancy outcomes, in face of the global threat of climate change.

## Appendix

**Table A1:** Studies examined for the meta analysis of the influence of month of conception on preeclampsia incidence.

Reference	Sample size	Setting	Tropical setting	Resolution	Hemisphere	Reason for exclusion from meta-analysis
[14]	79,298	WA (entire state), USA	No	Month	North	N.A.
[15]	7,904	Burlington, VT, USA	No	Month	North	N.A.
[16]	424,732	New South Wales, Australia	No	Month	South	N.A.
[18]	15,402	Hong Kong, China	No	Month	North	N.A.
[17]	2,824	Urmia, Iran	No	Month	North	N.A.
[19]	7,013	Bangkok, Thailand	Yes	2 seasons	North	Sole study in tropical climate

**Table A2:** Pooled relative risks and 95% credible interval for the association between month of conception on preeclampsia incidence after excluding [16].

Month in North hemisphere	January	February	March	April	May	June	July	August	September	October	November	December
Month in South hemisphere	July	August	September	October	November	December	January	February	March	April	May	June
Pooled relative risk	1 (ref)	1.16	1.01	1.12	1.17	1.33	1.15	1.22	1.12	1.02	0.96	1.06
Lower bound for 95% credible interval		1.1	0.57	0.89	0.98	0.78	0.67	0.89	0.97	0.79	0.7	0.84
Higher bound for 95% credible interval		1.22	1.79	1.43	1.4	2.27	1.97	1.65	1.3	1.31	1.31	1.33

**Table A3:** Studies examined for the meta analysis of the influence of month of birth on preeclampsia incidence.

Reference	Sample size	Setting	Tropical setting	Resolution	Hemisphere	Reason for exclusion from meta-analysis
[27]	18,500	Tel Aviv, Israel	No	Month	North	NA
[29]	28,262	Safata, Kuwait	No	Month	North	NA
[44]	3,050	Tulsa, OK, USA	No	Month	North	NA
[47]	203,462	Negev, Israel	No	Month	North	NA
[64]	11,979	Negev, Israel	No	Month	North	NA
[22]	312,207	TX (entire state), USA	No	Month	North	NA
[21]	1,869,388	Norway (entire country)	No	Month	North	NA
[48]	39,710	USA (12 cities) (12 hospitals)	No	Month	North	NA
[28]	11,585	Cape Town, South Africa	No	Month	South	NA
[20]	54,744	Southern, Zimbabwe	Yes	Month	South	NA

**Table A4:** Pooled relative risks and 95% credible interval for the association between month of birth on preeclampsia incidence after excluding [21].

Month in North hemisphere	January	February	March	April	May	June	July	August	September	October	November	December
Month in South hemisphere	July	August	September	October	November	December	January	February	March	April	May	June
Pooled relative risk	1.14	1.07	1.12	1.08	1.02	1.1	1 (ref)	0.96	0.98	1.04	1.08	1
Lower bound for 95% credible interval	0.83	0.67	0.76	0.64	0.65	0.65		0.56	0.6	0.7	0.77	0.56
Higher bound for 95% credible interval	1.56	1.7	1.64	1.8	1.61	1.85		1.66	1.61	1.54	1.53	1.77

**Table A5:** Studies examined for the meta analysis of the influence of season of birth on preeclampsia incidence.

Reference	Sample size	Setting	Tropical setting	Resolution	Hemisphere	Reason for exclusion from meta-analysis
[22]	312,207	TX (entire state), USA	No	4 seasons	North	NA
[15]	7,904	Burlington, VT, USA	No	4 seasons	North	NA
[31]	12,142	Rasht, Iran (NT),	No	4 seasons	North	NA
[41]	10,659	Uppsala, Sweden	No	4 seasons	North	NA
[29]	28,262	Safata, Kuwait	No	Month	North	NA
[44]	3,050	Tulsa, OK, USA	No	Month	North	NA
[30]	11,979	Negev, Israel	No	Month	North	NA
[46]	636	Tehran, (Iran),	No	4 seasons	North	NA
[45]	4,976	Zahedan, (Iran),	No	4 seasons	North	Unusable information
[23]	11,958	Jackson, MS, USA	No	3 seasons	North	No other study based on 3 seasons
[19]	7,013	Bangkok, Thailand	yes	2 seasons	North	Only 2 studies based on dry *vs*. rainy season with sufficient information
[24]	29,562	Mumbai, India	yes	2 seasons	North	Only 2 studies based on dry *vs*. rainy season with sufficient information
[25]	1,579	Enugu, Southern Nigeria	yes	2 seasons	North	No information on the seasonal distribution of the reference population
[20]	54,744	Southern, Zimbabwe	Yes	Month	South	Not enough information available to group by season

**Table A6:** Pooled relative risks and 95% credible interval for the association between season of birth on preeclampsia incidence.

Season		Winter	Spring	Summer	Fall
Pooled relative risk		1.05	1.05	1 (ref)	1.01
Lower bound for 95% credible interval		0.91	0.87		0.89
Higher bound for 95% credible interval		1.21	1.27		1.15

**Table A7:** Pooled relative risks and 95% credible interval for the association between season of birth on preeclampsia incidence after excluding [22].

Season		Winter	Spring	Summer	Fall
Pooled relative risk		1.11	1.26	1 (ref)	1.14
Lower bound for 95% credible interval		0.82	1.05		1.01
Higher bound for 95% credible interval		1.5	1.5		1.29

**Table A8:** Studies examined for the meta analysis of the association between temperature and preeclampsia incidence.

Reference	Sample size	Setting	Definition of temperature indicator	Temporal resolution for temperature indicator
[29]	28,262	Safata, Kuwait	Daily mean temperature	Month of birth
[28]	11,585	Cape Town, South Africa	Daily minimum and maximum temperatures	Month of birth
[30]	11,979	Beer Sheva, Israel	Daily maximum temperature	Month of birth
[31]	12,142	Rasht, Iran	Daily mean temperature	Season of birth (4)
[23]	11,958	Jackson, MS, USA	Daily maximum temperature	Season of birth (3)
[19]	7,013	Bangkok, Thailand	Daily maximum temperature	Season of birth (2)
[24]	29,562	Mumbai, India	Daily mean temperature	Season of birth (2)
[18]	15,402	Hong Kong, China	Heat Index (function of temperature and humidity)	Time of conception
[26]	4,400	Quebec, Canada	Grouped extreme high and low temperatures	20 first weeks of pregnancy
[27]	18,500	Tel Aviv, Israel	Average temperature	Month of birth

**Table A9:** Studies examined for the meta analysis of the influence of month of birth on eclampsia incidence.

Reference	Sample size	Location	Tropical	Resolution	Hemisphere	Reason for exclusion from meta-analysis
[40]	37,469	Maputo City, Mozambique	No	Month	South	NA
[39]	18,483	Karachi, Rawalpindi, Peshawar, and Quetta, Pakistan	No	Month	North	NA
[34]	482,759	Sweden (entire country)	No	Month	North	NA
[34]	12,170	Rajasthan, India	Yes	Month	North	NA
[37]		Abuja, Nigeria	Yes	Month	North	No control population
[38]	10,301	Accra, Ghana	Yes	Month	North	Study conducted in a setting with relative trends in month-to-month temperature changes markedly different from other studies

**Table A10:** Pooled relative risks and 95% credible interval for the association between month of birth on eclampsia incidence after excluding [34].

Month in North hemisphere	January	February	March	April	May	June	July	August	September	October	November	December
Month in South hemisphere	July	August	September	October	November	December	January	February	March	April	May	June
Pooled relative risk	3.47	2.49	1.55	1.89	1.83	1.98	1 (ref)	2.22	2.21	2.16	2.20	3.33
Lower bound for 95% credible interval	0.24	0.21	0.23	0.18	0.62	0.40		0.42	0.24	0.21	0.23	0.31
Higher bound for 95% credible interval	50.20	29.08	10.17	19.73	5.40	9.85		11.64	20.31	21.92	21.18	35.48

**Table A11:** Studies examined for the meta analysis of the influence of season of birth on eclampsia incidence.

Reference	Sample size	Location	Tropical	Resolution	Hemisphere	Reason for exclusion from meta-analysis
[49]	12,722	Thessaloniki, Greece	No	4 seasons	North	NA
[40]	37,469	Maputo City, Mozambique	No	Month	South	NA
[39]	18,483	Karachi, Rawalpindi, Peshawar, and Quetta , Pakistan	No	Month	North	NA
[34]	482,759	Sweden (entire country)	No	Month	North	NA
[36]	23,000	Peshawar, Pakistan	No	4 seasons	North	No reference at risk pregnancy number
[35]	Unknown	Hyderabad, India	Yes	Month	North	No sample size provided
[24]	29,562	Mumbai, India	Yes	2 seasons	North	No other tropical study available for an analysis of dry *vs*. rainy season
[38]	10,301	Accra, Ghana	Yes	Month	North	Study conducted in a setting with relative trends in month-to-month temperature changes markedly different from other studies

**Table A12:** Pooled relative risks and 95% credible interval for the association between season of birth on eclampsia incidence.

Season	Winter	Spring	Summer	Fall
Pooled relative risk	1.94	1.10	1 (ref)	1.14
Lower bound for 95% credible interval	1.19	0.90		0.79
Higher bound for 95% credible interval	3.16	1.33		1.66

**Table A13:** Pooled relative risks and 95% credible interval for the association between season of birth on eclampsia after excluding [34].

Season	Winter	Spring	Summer	Fall
Pooled relative risk	1.66	1.06	1 (ref)	1.28
Lower bound for 95% credible interval	0.43	0.61		0.46
Higher bound for 95% credible interval	6.32	1.83		3.56

**Table A14:** Studies examined for the meta analysis of the influence of season of conception on preterm birth and gestational length.

Outcome	Reference	Sample size	Country	Tropical	Resolution	Hemisphere
Preterm birth	[66]	291,517	Brandenburg and Saxony, Germany	No	4 seasons (OR)	North
Preterm birth	[75]	18,8276	NC (entire state), USA	No	4 seasons	North
Preterm birth	[51]	83,059	Pittsburgh, PA, USA	No	Month	North
Gestational length	[50]	1,435,213	3 US cities	No	Month	North

**Table A15:** Pooled relative risks and 95% credible interval for the association between season of conception and preterm birth.

Season	Winter	Spring	Summer	Fall
Pooled relative risk	1	1.05	1 (ref)	0.98
Lower bound for 95% credible interval	0.89	0.97		0.92
Higher bound for 95% credible interval	1.13	1.14		1.04

**Table A16:** Studies examined for the meta analysis of the influence of month of birth on preterm birth and gestational length.

Reference	Sample size	Location	Tropical	Resolution	Hemisphere	Reason for exclusion from meta-analysis
[64]	11,979	Negev, Israel	No	Month	North	N.A.
[77]	321,7738	Greece (entire country)	No	Month	North	N.A.
[66]	29,1517	Brandenburg and Saxony, Germany	No	Month	North	N.A.
[76]	7,665,006	Japan (entire country)	No	Month	North	N.A.
[56]	52,041,052	USA (entire country)	No	Month	North	N.A.
[58]	1,916	Keneba, Manduar, and Kantong Kunda, The Gambia	Yes	Month	North	Sole tropical study

**Table A17:** Pooled relative risks and 95% credible interval for the association between month of birth and preterm birth after excluding [56].

Month in North hemisphere	January	February	March	April	May	June	July	August	September	October	November	December
Pooled relative risk	1.14	1.04	1.01	1.05	1.09	1.18	1.1	1.08	1 (ref)	1.02	1.13	1.2
Lower bound for 95% credible interval	1.12	0.95	0.95	1.02	1.04	1.06	1.02	1.03		0.99	1.09	1.18
Higher bound for 95% credible interval	1.15	1.14	1.07	1.09	1.14	1.31	1.2	1.13		1.05	1.18	1.21

**Table A18:** Studies examined for the meta analysis of the influence of season of birth on preterm birth or gestational length.

Outcome	Reference	Sample size	Location	Tropical	Resolution	Hemisphere	Reason for exclusion from meta-analysis
Preterm birth	[54]	516,874	Greece (entire country)	No	4 seasons	North	N.A.
Preterm birth	[66]	291,517	Brandenburg and Saxony, Germany	No	4 seasons	North	N.A.
Preterm birth	[76]	7,665,006	Japan (entire country)	No	month	North	N.A.
Preterm birth	[30]	11,979	Negev, Israel	No	month	North	N.A.
Preterm birth	[77]	3,217,738	Greece (entire country)	No	month	North	N.A.
Preterm birth	[52]	1,669	Harare, Zimbabwe	Yes	4 seasons	South	Seasons defined as early/late dry/rainy, not consistent with other included studies
Preterm birth	[32]	14,040	Lombok, Indonesia	Yes	2 seasons	South	Seasons definition as dry/rainy, not consistent with other included studies
Preterm birth	[57]	855,320	Québec, Canada	No	2 seasons	North	Seasons definition according to the presence/absence of ice storm
Gestational length	[53]	16,796,415	Japan (entire country)	No	month	North	
Gestational length	[54]	516,874	Greece (entire country)	No	4 seasons	North	
Gestational length	[55]	1,166,206	Denmark (entire country)	No	4 seasons	North	Only 1 statement for results, lack of descriptive data
Gestational length	[52]	1,669	Harare, Zimbabwe	Yes	2 seasons	South	Seasonal definitions too different between this study and the other ones

**Table A19:** Pooled relative risks and 95% credible interval for the association between season of birth and preterm birth.

Season	Winter	Spring	Summer	Fall
Pooled relative risk	1.01	0.94	1 (ref)	0.94
Lower bound for 95% credible interval	0.90	0.83		0.85
Higher bound for 95% credible interval	1.13	1.07		1.03

**Table A20:** Studies examined for the meta analysis of the influence of month of birth on birth weight.

Reference	Term birth	Sample size	Setting	Tropical	Resolution	Hemisphere
[81]	Yes	10,631	Warsaw, Poland	No	month	North
[80]	Yes	418,817	Northern, Ireland	No	month	North
[79]	Yes	4,968,912	Chile (entire country)	No	month	South
[87]	Yes	24,325	12 US cities	No	4 seasons	North
[89]	Yes	19,783	Morogoro, Tanzania	Yes	2 seasons	South
[53]	No	16,796,415	Japan (entire country)	No	month	North
[82]	No	12,150	Aberdeen, Scotland	No	month	North
[56]	No	52,041,052	USA (entire country)	No	month	North
[83]	No	225,545	Israel (entire country)	No	month	North
[85]	No	4,507	Kimberly, Australia	No	month	North
[84]	No	1,573,203	Queensland, Australia	No	month	North
[86]	No	5,291	Rome, Italy and Sassary, Italy	No	4 seasons	North
[54]	No	516,874	Greece (entire country)	No	4 seasons	North
